# Stable pantothenamide bioisosteres: novel antibiotics for Gram-positive bacteria

**DOI:** 10.1038/s41429-019-0196-6

**Published:** 2019-06-06

**Authors:** Patrick A. M. Jansen, Danique A. van der Krieken, Peter N. M. Botman, Richard H. Blaauw, Lorenzo Cavina, Eline M. Raaijmakers, Erik de Heuvel, Julia Sandrock, Lian J. Pennings, Pedro H. H. Hermkens, Patrick L. J. M. Zeeuwen, Floris P. J. T. Rutjes, Joost Schalkwijk

**Affiliations:** 1 Department of Dermatology, Radboud University Medical Center, Nijmegen, The Netherlands; 2 Chiralix, Nijmegen, The Netherlands; 3 Department of Medical Microbiology, Radboud University Medical Center, Nijmegen, The Netherlands; 4 Hermkens Pharma Consultancy, Oss, The Netherlands; 5 Institute for Molecules and Materials, Radboud University, Nijmegen, The Netherlands

**Keywords:** Antibiotics, Drug discovery and development

## Abstract

The emergence of multidrug resistant bacteria has prioritized the development of new antibiotics. *N*-substituted pantothenamides, analogs of the natural compound pantetheine, were reported to target bacterial coenzyme A biosynthesis, but these compounds have never reached the clinic due to their instability in biological fluids. Plasma-stable pantothenamide analogs could overcome these issues. We first synthesized a number of bioisosteres of the prototypic pantothenamide N7-Pan. A compound with an inverted amide bond (CXP18.6-012) was found to provide plasma-stability with minimal loss of activity compared to the parent compound N7-Pan. Next, we synthesized inverted pantothenamides with a large variety of side chains. Among these we identified a number of novel stable inverted pantothenamides with selective activity against Gram-positive bacteria such as staphylococci and streptococci, at low micromolar concentrations. These data provide future direction for the development of pantothenamides with clinical potential.

## Introduction

**Fig. 1:**
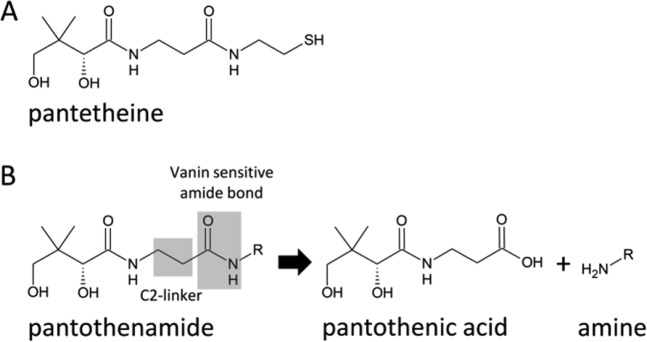
General structures: **a** Chemical structure of pantetheine. **b** General structure of pantothenamide and its products pantothenic acid and the corresponding amine after hydrolysis by vanins. The vanin sensitive amide bond is marked as well as the C2-linker between the amides

There is an ongoing need for new drug scaffolds and targets to combat the increasing development of resistant bacterial strains. Methicillin-resistant and vancomycin-resistant *Staphylococcus aureus* (*S. aureus*) strains (MRSA and VRSA) have led to serious health problems in both hospital settings and the community, but resistance to antibiotics is certainly not limited to these examples. Pantothenate or pantothenic acid (vitamin B5) is required for coenzyme A (CoA) biosynthesis and is an essential and rate limiting nutrient for survival and/or growth of numerous bacteria, fungi and protozoa. A range of compounds analogous to pantothenate or to its cysteamine conjugate pantetheine (see Fig. 1a) have been reported that possess activity against bacteria, fungi and malaria parasites (for a review see ref. [1]). In 1970, *N*-substituted pantothenamides (in short: pantothenamides), were first reported to possess antibacterial activity in vitro(see Fig. 1b for the general pantothenamide structure) [2]. Pantothenamides are pantetheine analogs, of which the prototypic members *N*-pentylpantothenamide (N5-Pan) and *N*-heptylpantothenamide (N7-Pan) were found to be active against Gram-negative and Gram-positive bacteria [3]. During the last few decades, a number of additional pantothenamides with antibacterial activity have been synthesized [4–8] and their putative modes of action have been studied in detail [7–11]. Pantothenamides have been shown to serve as substrates or inhibitors (either competitive or allosteric) of pantothenate kinase (PanK), the first enzyme in the CoA biosynthesis pathway [4, 5, 7, 8]. As a consequence, PanK-catalyzed pantothenate phosphorylation is partially or completely inhibited. In the case that pantothenamides serve as PanK substrates, competing with the natural substrate pantothenic acid, the resulting 4′-phosphopantothenamides may be further metabolized by the CoA biosynthetic machinery to yield analogs of CoA, as was shown for *Escherichia coli (E. coli)* [9, 10]. Such CoA analogs were found to be incorporated in acyl carrier protein, thereby inhibiting its function in bacterial fatty acid biosynthesis, which requires the 4′-phosphopantetheine moiety of CoA to be active [12]. Whether the mechanisms that ultimately result in antimicrobial activity in the various target organisms (bacteria, fungi, protozoa) are the result of inhibition of CoA biosynthesis, fatty acid biosynthesis or another CoA-utilizing process, or a combination of the above, remains to be resolved for most organisms. The mechanism of action in *S. aureus*, however, has recently been investigated in detail indicating that PanK is the target enzyme [7].

Despite their potential use and selectivity for bacterial, fungal, and/or protozoan metabolic routes, no pantothenamide compound has ever made it to the clinic. It was recently shown that pantothenamides are not active as antimicrobials in the presence of serum, and that they were hydrolyzed by ubiquitous pantetheinases of the vanin family (see Fig. 1b) [13–16]. To address this, a series of vanin inhibitors based on a pantothenate scaffold were synthesized, which proved to inhibit serum vanin activity in the nanomolar range [17]. Combinations of these novel vanin inhibitors and prototypic pantothenamides like N5-Pan and N7-Pan exert antimicrobial activity in vitro, particularly against Gram-positive bacteria (*S. aureus*, *Staphylococcus epidermidis* (*S. epidermidis*), *Streptococcus pneumonia* (*S. pneumoniae*), and *Streptococcus pyogenes* (*S. pyogenes*)) even in the presence of serum [18]. These results indicate that pantothenamides, when protected against degradation by host vanins, are potentially useful antimicrobial agents [15, 16].

As combinations of compounds are less desirable from a drug development perspective, chemical modification of pantothenamides to render them more stable would be preferable. Taking this approach, modified pantothenamides were generated that were active antimalarials or antibacterials in the presence of serum [19–23]. As the hydrolysis-susceptible amide bond was still present in these modifications or the potency was reduced significantly, we aimed to generate bioisosteric pantothenamides that would mimic the chemical space of the amide bond, but would lack the vanin sensitivity, without losing their potency. Here we describe such stable bioisosteric compounds as a starting point for lead optimization.

## Materials and methods

### Chemistry of inverted pantothenamides

#### General procedure A

To a solution of carboxylic acid **B** (0.5 mmol) in MeCN/H_2_O (30:1, 4.3 ml) were added HOBt (0.6 mmol), NaHCO_3_ (0.6 mmol), EDCI (0.6 mmol), and a solution of amine **A** (for the synthesis see ref 2, 0.6 mmol) in MeCN/H_2_O (0.7 ml). The progress of the reaction was monitored using LC-MS and upon completion, the reaction was quenched by the addition of saturated aqueous NH_4_Cl solution (15 ml) and the mixture was extracted twice using EtOAc (15 ml). The combined organic layers were dried over Na_2_SO_4_ and filtered before concentration under reduced pressure. The residue was purified by flash column chromatography (DCM/MeOH = 98:2 → 80:20) to afford the product.



#### CXP18.6-012

According to general procedure A. Yield: 24%, white solid. ^1^H NMR (400 MHz, CDCl_3_): *δ* 7.23 (br s, 1H), 6.15 (br s, 1H), 4.01 (s, 1H), 3.84 (br s, 1H), 3.55–3.34 (m, 6H), 3.22 (br s, 1H), 2.20–2.14 (m, 2H), 1.70–1.55 (m, 2H), 1.35–1.20 (m, 8H), 1.04 (s, 3H), 0.96 (s, 3H), 0.88 (t, *J* = 6.9 Hz, 3H).

#### CXP18.6-013

According to general procedure A. Yield: 40%, white solid. ^1^H NMR (400 MHz, CDCl_3_): *δ* 7.28 (br s, 1H), 6.26 (br s, 1H), 4.10-3.97 (m, 2H), 3.55–3.30 (m, 7H), 2.21–2.13 (m, 2H) 1.65–1.55 (m, 2H), 1.38–1.22 (m, 4H), 1.03 (s, 3H), 0.95 (s, 3H), 0.88 (t, *J* = 7.0 Hz, 3H).

#### CXP18.6-014

According to general procedure A. Yield: 66%, white solid. ^1^H NMR (400 MHz, CDCl_3_): *δ* 7.27 (br s, 1H), 6.23 (br s, 1H), 4.03–3.98 (m, 2H), 3.53–3.33 (m, 7H), 2.20–2.14 (m, 2H), 1.65–1.54 (m, 2H), 1.34–1.20 (m, 12H), 1.03 (s, 3H), 0.95 (s, 3H), 0.88 (t, *J* = 6.9 Hz, 3H).

#### CXP18.6-017

According to general procedure A. Yield: 35%, colorless oil. ^1^H NMR (400 MHz, CD_3_OD): *δ* 7.28–7.17 (m, 2H), 7.11–6.98 (m, 2H), 3.88 (s, 1H), 3.46 (d, *J* = 11.0 Hz, 1H), 3.39 (d, *J* = 11.0 Hz, 1H), 3.33–3.22 (m, 4H), 2.98–2.92 (m, 2H), 2.51–2.54 (m, 2H), 0.92 (s, 3H), 0.92 (s, 3H).

#### CXP18.6-069

According to general procedure A. Yield: 43%, colorless oil. ^1^H NMR (400 MHz, CD_3_OD): *δ* 7.30–7.23 (m, 1H), 6.93–6.83 (m, 2H), 3.88 (s, 1H), 3.46 (d, *J* = 11.0 Hz, 1H), 3.39 (d, *J* = 11.0 Hz, 1H), 3.33–3.22 (m, 4H), 2.92 (app t, *J* = 7.7 Hz, 2H), 2.48–2.42 (m, 2H), 0.92 (s, 3H), 0.92 (s, 3H).

#### CXP18.6-047

According to general procedure A. Yield: 25%, colorless oil. ^1^H NMR (400 MHz, CD_3_OD): *δ* 7.18 (dd, *J* = 5.1, 1.2 Hz, 1H), 6.90 (dd, *J* = 5.1, 3.4 Hz, 1H), 6.86–6.81 (m, 1H), 3.87 (s, 1H), 3.46 (d, *J* = 11.0 Hz, 1H), 3.39 (d, *J* = 11.0 Hz, 1H), 3.35–3.23 (m, 4H), 3.17–3.09 (m, 2H), 2.56–2.49 (m, 2H), 0.92 (s, 3H), 0.92 (s, 3H).

#### CXP18.6-057

According to general procedure A. Yield: 48%, off-white solid. ^1^H NMR (400 MHz, CD_3_OD): *δ* 7.35 (dd, *J* = 1.9, 0.8 Hz, 1H), 6.28 (dd, *J* = 3.2, 1.9 Hz, 1H), 3.89 (s, 1H), 3.46 (d, *J* = 11.0 Hz, 1H), 3.39 (d, *J* = 11.0 Hz, 1H), 3.36–3.25 (m, 4H), 2.96–2.86 (m, 2H), 2.55–2.45 (m, 2H), 0.93 (s, 3H), 0.92 (s, 3H).

#### CXP18.6-064

According to general procedure A. Yield: 92%, colorless oil. ^1^H NMR (400 MHz, CD_3_OD): *δ* 7.10 (app t, *J* = 7.9 Hz, 1H), 6.92–6.83 (m, 2H), 3.88 (s, 1H), 3.46 (d, *J* = 11.0 Hz, 1H), 3.39 (d, *J* = 11.0 Hz, 1H), 3.33–3.22 (m, 4H), 2.94–2.85 (m, 2H), 2.48–2.40 (m, 2H), 2.30 (s, 3H), 0.92 (s, 3H), 0.91 (s, 3H).

#### CXP14.18-005

According to general procedure A. Yield: 25%, colorless oil. ^1^H NMR (400 MHz, CDCl_3_): *δ* 7.30 (br s, 1H), 6.32 (br s, 1H), 4.02 (s, 1H), 3.51 (s, 2H), 3.49–3.34 (m, 4H), 2.22–2.15 (m, 2H), 1.62–1.46 (m, 3H), 1.03 (s, 3H), 0.96 (s, 3H), 0.91 (s, 3H), 0.89 (s, 3H).

#### CXP14.18-012

According to general procedure A. Yield: 88%, white solid. ^1^H NMR (400 MHz, CDCl_3_): *δ* 7.28 (br s, 1H), 6.26 (br s, 1H), 4.01 (s, 1H), 3.51 (s, 2H), 3.50–3.31 (m, 4H), 2.20–2.14 (m, 2H), 1.65–1.55 (m, 2H), 1.37–1.18 (m, 16H), 1.30 (s, 3H), 0.95 (s, 3H), 0.88 (t, *J* = 6.9 Hz, 3H).

#### CXP14.26-007

According to general procedure A. Yield: 28%, white waxy solid. ^1^H NMR (400 MHz, CD_3_OD): *δ* 3.98 (dd, *J* = 8.0, 3.9 Hz, 1H), 3.89 (s, 1H), 3.47 (ABd, *J* = 10.9 Hz, 1H), 3.40 (ABd, *J* = 10.9 Hz, 1H), 3.40–3.30 (m, 4H), 1.81–1.70 (m, 1H), 1.63–1.52 (m, 1H), 1.46–1.27 (m, 4H), 1.0–0.85 (m, 9H).

#### CXP14.18-037

Amine **A** (1.50 g, 7.88 mmol) was dissolved in acetone (25 ml) and cooled to 4 °C. 2-Methoxyprop-1-ene (2.26 ml, 23.6 mmol) and TsOH (1.65 g, 8.67 mmol) were added and after 15 min the cooling bath was removed. The reaction was stirred for 18 h and then quenched by the addition of Et_3_N (1.60 g, 15.7 mmol), followed by 12.5% aqueous ammonia (15 ml). The mixture was extracted twice with DCM (15 ml) and the combined organic layers were concentrated under reduced pressure. The residue was purified by flash column chromatography (EtOAc/MeOH/25% aq. NH_4_OH = 90:10:1 → 50:50:1) to afford 1.07 g amine **C** as a colorless oil. Yield: 30%. ^1^H NMR (400 MHz, CD_3_OD): *δ* 4.15 (s, 1H), 3.75 (ABd, *J* = 11.7 Hz, 1H), 3.24–3.30 (m, 3H), 2.73 (m, 2H), 1.46 (s, 3H), 1.45 (s, 3H), 1.00 (s, 3H), 0.99 (s, 3H).

To a solution of 2-(propylthio)acetic acid (0.15 g, 0.81 mmol) in a mixture of MeCN (4.4 ml) and H_2_O (0.24 ml) were added HOBt (0.11 g, 0.71 mmol), DIPEA (0.14 ml, 0.81 mmol), EDC (0.14 g, 0.72 mmol) and amine **C** (0.15 g, 0.65 mmol). The reaction was stirred at room temperature for 2 h, before the addition of EtOAc (10 ml). The mixture was washed with saturated aqueous NH_4_Cl (10 ml), and saturated aqueous NaHCO_3_ (10 ml), dried over Na_2_SO_4_ and filtered before concentration under reduced pressure. The residue was purified by flash column chromatography (EtOAc/MeOH = 95:5 → 90:10) to afford amide **D** (147 mg, 65%).

To a solution of amide **D** (0.15 g, 0.42 mmol) in MeCN (2.1 ml) was added an aqueous 0.2 M solution of HCl (2.12 ml, 0.42 mmol). The mixture was stirred at room temperature for 2 h. The mixture was added dropwise to a mixture of saturated aqueous NaHCO_3_ (2 ml) and EtOAc (10 ml). The layers were separated and the aqueous phase was saturated with NaCl and then extracted with EtOAc (10 ml). The combined organic layers were dried over Na_2_SO_4_ and filtered before concentration under reduced pressure to afford **CXP14.18-037** (113 mg, 85%) as a pale yellow oil. ^1^H NMR (400 MHz, CDCl_3_): *δ* 7.30 (br s, 1H), 6.30 (br s, 1H), 4.13 (d, *J* = 5.0 Hz, 1H), 4.01 (d, *J* = 5.0 Hz, 1H), 3.55–3.33 (m, 7H), 2.22–2.15 (m, 2H), 1.65–1.53 (m, 2H), 1.39–1.25 (m, 2H), 1.03 (s, 3H), 0.95 (s, 3H), 0.91 (t, *J* = 7.3 Hz, 3H).



#### CXP14.18-028

According to the sequence used for CXP14.18-037. Yield (from **C**): 55%, colorless oil. ^1^H NMR (400 MHz, CD_3_OD): *δ* 3.89 (s, 1H), 3.47 (d, *J* = 10.9 Hz, 1H), 3.39 (d, *J* = 10.9 Hz, 1H), 3.40–3.28 (m, 4H), 2.21–2.15 (m, 2H), 1.65–1.53 (m, 2H), 1.44–1.29 (m, 2H), 0.94–0.91 (m, 9H).

#### CXP14.18-034

According to the sequence used for CXP14.18-037. Yield (from **C**): 37%, pale yellow oil. ^1^H NMR (400 MHz, CDCl_3_): *δ* 7.25 (br s, 1H), 6.32 (br s, 1H), 4.02 (d, *J* = 5.1 Hz, 1H), 3.97 (d, *J* = 5.1 Hz, 1H), 3.55–3.27 (m, 7H), 2.53 (t, *J* = 7.0 Hz, 2H), 2.31 (t, *J* = 7.4 Hz, 2H), 2.09 (s, 3H), 1.99–1.87 (m, 2H), 1.03 (s, 3H), 0.95 (s, 3H).

#### CXP14.18-038

According to the sequence used for CXP14.18-037. Yield (from **C**): 34%, colorless oil. ^1^H NMR (400 MHz, CDCl_3_): *δ* 7.28 (br s, 1H), 6.44 (br s, 1H), 4.03–3.93 (m, 1H), 3.55–3.33 (m, 7), 2.90–2.70 (m, 2H), 2.55 (q, *J* = 7.8 Hz, 2H), 2.53–2.38 (m, 2H), 1.26 (t, *J* *=* 7.8 Hz, 3H), 1.04 (s, 3H), 0.95 (s, 3H).

### Bacterial strains, growth conditions, and MIC assay

The following bacterial strains were used in this study. *S. aureus* (ATCC6538, ATCC29213, Xen36, and MRSA (clinical isolate kindly provided from RIVM)), *S. epidermidis* (ATCC12228, ATCC14990 and Bactimm 389 (clinical isolate provided from Bactimm)), *S. pyogenes* (SS91, SS410 and SS799), *Escherichia coli* (ATCC25922), *Pseudomonas aeruginosa* (ATCC15692), *Mycobacterium avium* (ATCC700898), *Mycobacterium abscessus* (CIP104536), and *Mycobacterium kansasii* (ATCC25221).

All strains, except mycobacteria, were grown overnight on Columbian blood agar plates (Thermo Scientific) at 37 °C. Slow growing mycobacteria (SGM, *M. avium* and *M. kansasii*) were grown for 5–7 days in Middlebrook 7H9 Broth supplemented with 10% oleic acid, albumin, dextrose, and catalase (OADC) at 37 °C. Rapid growing mycobacteria (RGM, *M. abscessus*) were grown for 3 days in Middlebrook 7H9 Broth at 30 °C. *S. pyogenes* strains were incubated at 5% CO_2_, while all other strains grow in normal atmosphere. Liquid cultures of *S. aureus*, *S. epidermidis*, *E. coli*, and *P.aeruginosa* were grown in Mueller-Hinton Broth (BD Difco) at 37 °C while shaking and *S. pyogenes* were grown statically in 5% CO_2_ at 37 °C in Todd Hewitt Broth (BD Bacto).

To test the MIC of compounds on *S. aureus*, *S. epidermidis*, *S. pyogenes*, *E. coli*, and *P. aeruginosa* overnight cultures were diluted 1:1000 in fresh media and 100 µl was added to 100 µl of serial diluted compound in 96-well plates. Plates were incubated at 37 °C (*S. pyogenes* in 5% CO_2_, others in normal atmosphere) for 16 h and MICs were determined optically. The MIC was defined as the first well where no bacterial growth was observed. To test the MIC of compounds on *M. avium, M. kansasii,* and *M. abscessus*, a 0.5 McFarland suspension was made of the cultured strains during log phase growth. This suspension was then diluted to obtain an inoculum of 1 × 10^5^–1 × 10^6^ CFU ml^−1^ in Cation Adjusted Mueller-Hinton broth (with 20% OADC for SGM) and 50 µl was added to 50 µl of serial diluted compound in 96-well plates. Plates were incubated at 37 °C for 7 days for SGM and at 30 °C for 3 days for RGM. MICs were determined optically. The MIC was defined as the first well where no bacterial growth was observed. The results are the median values of at least three independent assays.

Antimicrobial activity of N7-Pan and CXP18.6-012 in the presence of serum was performed as follows: to mimic human serum as much as possible, the highest concentration of serum (10%) was used that did not interfere with the optical readout. Complement inactivation of human serum (30 min at 56 °C) was necessary to prevent antimicrobial activity of the complement system. Because heat inactivation of serum also led to decreased vanin activity, we supplemented the serum with fetal bovine serum (FBS) in a ratio of 4:1. The vanin activity in FBS is extremely high compared to human serum, leading to a total vanin activity in the assay that resembles full human serum. Furthermore, the standard MIC assay was performed as described above.

### LC-MS analysis

To examine the stability of analogs in vitro, N5-Pan, its bioisostere CXP18.6-013, N7-Pan and its bioisostere CXP18.6-12 were incubated for 16 h at room temperature in 500 µM potassium buffer with and without 10% fetal bovine serum as a source of pantetheinase activity. Samples were taken and analyzed by LC-MS using a Shimadzu LC10ATvp HPLC coupled to a Shimadzu LCMS2010A mass spectrometer. The elution of the following molecules were shown: N5-Pan and CXP18.6-013 (271 [M-H_2_O + H^+^]), N7-Pan and CXP18.6-012 (299[M- H_2_O + H^+^]), pantothenate (220 [M + H^+^]) and N2-(aminoethyl)−2,4-dihydroxy-3,3-dimethylbutyramide (191 [M + H^+^]).

### Cytotoxicity assay

Primary human keratinocytes obtained from biopsies of healthy volunteers were cultured following the Rheinwald-Green system at 37 °C and 5% CO_2_ [24]. Cells were grown until they reached confluency. Compounds were added to these cultures at a concentration of 100 µM and effect on growth and toxicity was determined microscopically after 24 h. Cytotoxicity was detected using the LDH cytotoxicity detection kit according to the manufacturer’s protocol (Roche Applied Science, IN).

## Results

### Synthesis of N7-pan bioisosteres

We investigated the structure-activity relationship of pantothenamide modifications by varying the N7-Pan molecule on three different positions, as indicated in Fig. 1b: the C2 linker between the two amides, the second amide bond and the right hand part of the molecule (further referred to as side chain). Table 1 lists the structural modifications in the C2 linker and amide bond of N7-Pan, and their effects on the minimal inhibitory concentration (MIC) values. It turned out that only a C2 linker was allowed. Substitution by an alkene or aromatic moiety was not allowed. The amide bond was probed by replacing with different bioisosteres, whilst keeping the C2 linker intact. Replacement by a keto, ester or a sulfonamide moiety was not allowed. Replacement by 5-membered ring amide bioisosteres was in general not successful, although in some cases minor activity was demonstrated. The only effective modification which yielded activities comparable to the parent compound was substitution of the amide bond by an inverted amide as shown in compound CXP18.6-012. Like N7-Pan, none of its bioisosteres showed activity against the Gram-negative bacteria that were tested (*E. coli* and *P. aeruginosa*) or the *Mycobacterium* species that were tested (*M. avium*, *M. abscessus* and *M. kansasii*). Some bioisosteres showed weak activities against *S. epidermidis* and *S. pyogenes* (MIC between 8 and 32 μg ml^−1^). Only bioisostere CXP18.6-012 showed activity against *S. aureus*, with a MIC value of 2 μg ml^−1^, which is close to the potency of the parent compound N7-Pan. However, this modification decreased the potency towards *S. epidermidis* from 0.5 to 8 μg ml^−1^, whereas the sensitivity towards *S. pyogenes* was decreased from 2 to 32 μg ml^−1^. In addition, we synthesized the inverted amide bioisostere of another prototypical pantothenamide, N5-Pan, designated as CXP18.6-013 (see also Supplemental Table 1). This compound showed good activity towards *S.pyogenes* (2 μg ml^−1^) and weak activity towards *S. epidermidis* and *S.aureus* (resp. 16 and 32 μg ml^−1^). The experimental details on the synthesis of the compounds of Table 1 are given in online Supplemental File S1, with the exception of CXP18.6-012 which can be found in the Materials and Methods section, and N7-Pan, which has been described before [2].

**Table 1:** Bioisosteres of prototypic pantothenamide N7-Pan

MICs were denoted as µg ml^−1^

MICs up to 32 µg ml^−1^ are represented in bold

### Stability of inverted pantothenamides

**Fig. 2:**
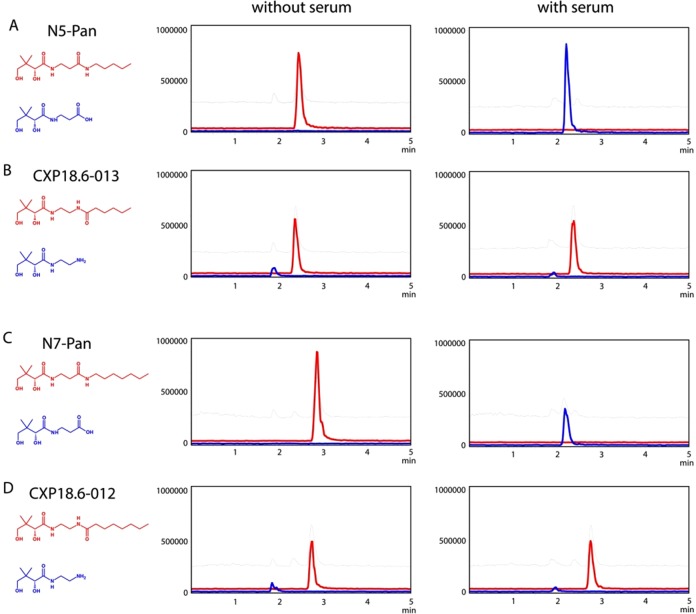
Stability of inverted pantothenamides in serum. The stability of compounds was measured using LC-MS analysis after overnight incubation in buffer with or without 10% fetal bovine serum. Fetal bovine serum contains high levels of pantetheinase activity. **a** N5-Pan (red line) was stable in buffer, but completely degraded when serum was added. The blue peak represents pantothenate, the product after pantetheinase-derived degradation. **b** CXP18.6-013 (red line), the inverted amide of N5-Pan remain stable in serum-containing buffer, indicating it is protected against degradation by vanins. The hypothetical product upon degradation by pantetheinase activity (blue peak) was not detected. **c** N7-Pan (red line) was also sensitive for enzymatic degradation as described in **a**. **d** CXP18.6-012 (red line), the inverted amide from N7-Pan remained stable as described for CXP18.6-013 (**b**). Note that the small blue peak in (**b**) and (**d**) just before 2 min of retention is from another molecule of the same mass, probably an ingredient of the buffer. Total ion current was shown in gray at 1x intensity, while the other lines were shown at a 10x intensity

We incubated the prototypical pantothenamides N5-Pan and N7-Pan as well as their inverted amide bioisosteres CXP18.6-012 and CXP18.6-013 in the presence and absence of fetal bovine serum, and analyzed the stability towards vanin-mediated hydrolysis, using LC-MS analysis. In Fig. 2a, c total degradation of N5-Pan and N7-Pan by serum is shown as the disappearance of the red peak of the parent compound, whereas the hydrolysis product pantothenate emerges (blue peak). Figures 2b, d show that the inverted pantothenamides CXP18.6-013 and CXP18.6-012 remain stable after overnight incubation in a serum-containing buffer.

### Activity of inverted pantothenamides under physiological conditions

We then investigated whether inverted pantothenamides would retain their ability to kill bacteria, even in the presence of serum. To test this, we performed MIC assays in Mueller-Hinton medium to which 10% serum was added. We tested both human serum and fetal bovine serum (FBS). Human serum was first decomplemented at 56 °C, which also partly destroys its vanin activity. FBS, which has an extremely high vanin activity, was used without heat inactivation, which allowed testing of the compounds in the presence of a vanin activity which equals that of 100% human serum. Table 2 shows that addition of serum abolished the antibiotic properties of N7-Pan, whereas CXP18.6-012 remained active.

**Table 2:** Effect of serum on MIC of N7-Pan and CXP18.6–012 against *S. aureus* ATCC6538

### Structural modification of the side chain of inverted N7-Pan

Having established that the inverted N7-Pan was active and stable, and knowing that variation of the C2 linker was not allowed, we aimed to improve its potency or alter the specificity by modification of the side chain, using the C2 linker a backbone and keeping the second amide bond inverted. Supplemental Table 1 lists all 72 side chain modifications and their activity against *S. aureus*, *S. epidermidis*, *S. pyogenes*, *E.coli*, *P. aeruginosa*, *M. avium*, *M. abscessus*, and *M. kansasii*. Both aliphatic and aromatic substitutions provided active compounds. With aliphatic side chains the optimum length was C7 and C9. In case of shorter chain length (C5, C5 with a heteroatom), activity was mainly observed against *S. pyogenes* (e.g., CXP18.6-013, CXP14.18-028, CXP14.18-037, and CXP14.18-034). It turned out that the C7 aliphatic chain could be replaced by ethylaromatics. In case of a phenyl being present in the side chain, a comparable activity profile was found (see CXP18.6-064, CXP18.6-017, and CXP18.6-069). In case of aromatic heterocycles, activity against *S. pyogenes* became dominant (CXP18.6-047 and CXP18.6-057). None of the tested inverted pantothenamides was able to inhibit growth of Gram-negative bacteria or *Mycobacterium* species at relevant concentrations (≤32 μg ml^−1^). The compounds that showed significant activity towards one of the Gram-positive bacteria were tested against multiple strains of *S. aureus*, *S. epidermidis*, and *S. pyogenes* (see Table 3). Overall, the differences between strains were very small. The most potent inverted pantothenamides against *S. aureus* tested here are CXP18.6-17 (1–2 μg ml^−1^), CXP18.6-012 (2–8 μg ml^−1^), CXP18.6-014 (2–8 μg ml^−1^), and CXP18.6-069 (2–8 μg ml^−1^). Towards *S. epidermidis* the most potent inverted pantothenamides were CXP18.6-064 (1 μg ml^−1^), CXP18.6-014 (2 μg ml^−1^), CXP18.6-69 (1–2 μg ml^−1^), and CXP18.6-17 (2–4 μg ml^−1^). Strains of *S.pyogenes* were most sensitive to CXP14.18-028 (2 μg ml^−1^), CXP14.18-037 (2–4 μg ml^−1^), and CXP14.18-005 (2–8 μg ml^−1^).

**Table 3:** MIC of active inverted pantothenamides on multiple strains

Compound	*S.aureus* ATCC6538	*S.aureus* ATCC29213	*S.aureus* MRSA	*S.aureus* Xen36	*S.epidermidis* ATCC12228	*S.epidermidis* Bactimm 389	*S.epidermidis* ATCC14990	*S.pyogenes* SS91	*S.pyogenes* SS410	*S.pyogenes* SS799
CXP18.6–012	2	2	8	4	8	4	4	32	>32	>32
CXP18.6–013	32	>32	>32	16	16	>32	16	2	2	2
CXP18.6–014	4	2	4	8	2	2	2	32	32	>32
CXP18.6–017	2	1	2	2	2	2	4	>32	>32	>32
CXP18.6–069	8	2	4	8	1	2	1	>32	>32	>32
CXP18.6–047	16	32	32	8	16	16	8	8	16	16
CXP18.6–057	16	16	32	8	8	16	8	4	8	4
CXP18.6–064	4	8	4	8	1	1	1	>32	>32	>32
CXP14.18–028	>32	>32	>32	>32	>32	16	8	2	2	2
CXP14.18–005	>32	>32	>32	>32	>32	32	8	8	2	4
CXP14.26–007	>32	>32	>32	>32	>32	>32	>32	8	8	8
CXP14.18–037	>32	>32	>32	32	32	>32	16	4	2	2
CXP14.18–038	>32	>32	>32	>32	>32	>32	32	8	4	8
CXP14.18–034	>32	32	>32	16	>32	32	8	8	4	8
CXP14.18–012	>32	8	32	32	2	4	2	16	8	16

MICs were denoted as μg ml^−1^. Structures are depicted in Supplemental Table 1.

### In vitro toxicology

Finally, we used cultured primary human keratinocytes to obtain limited in vitro toxicology data on inverted pantothenamides. Using LDH release as a measure of viability, we did not detect cytotoxicity of any of the compounds that showed antimicrobial activity up to concentrations of 100 μM (~30  μg ml^−1^).

## Discussion

In the present study we describe a new class of stable pantothenamide bioisosteres that display antibiotic activity against staphylococci and streptococci. These compounds target a biochemical pathway that is distinct from currently marketed drugs, and their activity appears to be primarily against clinically relevant Gram-positive species. As there is a strong medical need for improved small-spectrum antibiotics, stable pantothenamides with improved potency may be moved forward as promising compounds for clinical use.

Previous studies have demonstrated that pantothenamide antibiotics based on the pantothenate/pantetheine scaffold are labile in biological fluids due to hydrolysis of the amide bond by serum vanins [13–16]. Our data reveal that inversion of the amide bond afforded a considerable increase of stability whilst largely preserving the biological activity [25]. A recent paper by Barnard et al. [20] reported that the inversion of the amide bond had a dramatic effect on antibiotic activity towards the *S. aureus* RN2440 strain. This is in contrast to our findings. We have tested multiple strains and species, and found similar MICs among strains. The basis for the discrepancy between these studies is presently unclear, but is currently actively being pursued in collaboration with Strauss and co-workers. Other structural modifications, reported for antimalarial pantothenamides, that increased the stability include introduction of a methyl group in the C2 linker [19] and variation of the linker length [8]. We further explored structural modifications at the side chain, which did not lead to improved potency compared to the parent compound. Remarkably, the taxon-specificity was clearly dependent on the structural modifications in the molecule. These were found to affect both the specificity at the genus and the species level for Gram-positive organisms studied here. We do not think that the pantothenamides as a class are selective for Gram-positive organisms. In the original paper by Clifton et al[2]. where N5-Pan and N7-Pan were first described, there was significant activity of N5-Pan against the Gram-negative organism *E. coli*. Here we have developed compounds based on N7-Pan, which appear to be more potent towards Gram-positive organisms. It has to be noted that we only tested a number of clinically relevant genera of Gram-positive and Gram-negative bacteria. A more extensive screen of bacterial species is required to establish the sensitivity of other Gram-negative organisms. The observed lack of sensitivity of *E. coli* might be explained by the fact that N7-Pan (and possibly also its derivatives) is a substrate for TolC-dependent efflux pumps, as reported by Zhang et al. [10].

Our present study suggests that pantothenamides may be further optimized to yield small-spectrum antibiotics that could target a pathogen without affecting the complete human microbiome. This would be a major advance to avoid the consequences of broad spectrum antibiotics such gut microbiota dysbiosis, thereby reducing antibiotic-associated complications such as diarrhea, *Clostridium difficile* infections or candidiasis. An example that such specificity is feasible is the development of Debio 1450 (now known as afabicin), an antibiotic candidate with a novel mechanism of action, currently in phase II clinical development for intravenous and oral treatment of staphylococcal infections [26]. It specifically targets *Staphylococcus* by inhibition of FabI, an enzyme essential for fatty acid synthesis in this genus. Debio 1450 shows a high potency against staphylococci, particularly MRSA, without cross-resistance to other antibiotics, including other agents active against staphylococci. Interestingly, this indicates that bacterial fatty acid synthesis is a valid antibiotic target, at least for *S. aureus*, which has been questioned previously [27]. As CoA is also required for fatty acid synthesis, pantothenamides may also act on bacterial fatty acid synthesis. Addition of serum, which is a source of host fatty acids, to our MIC assays did not significantly inhibit the antibiotics effects. This indicates that exogenous available free fatty acids do not interfere with the mechanism of action.

The mechanism of action of the prototypic pantothenamides has been investigated previously and appears largely dependent on the PanK type of the organism. For *E.coli*, which has a PanK_I_ type enzyme, the presumed mechanism of action of the pantothenamide N5-pan has been investigated in detail. The formation of coenzyme A antimetabolites [9] that interferes with fatty acid synthetic enzymes [11] and endogenous pantothenate biosynthesis [28] are two affected pathways that are interrupted. It was also reported that N5-Pan could inhibit the growth of *S. pneumoniae* and that this effect was probably due to fatty acid synthesis inhibition as it could be outcompeted with oleate [10]. The molecular mechanism by which pantothenamides inhibit growth of other Gram-positive bacteria is currently unknown, with an exception for *S. aureus*. In contrast to other gram-positive bacteria, *S. aureus* has a PanK_II_ type enzyme that accepts pantothenamides as a substrate [8]. The co-crystal structures of SaPanK_II_ and N7-pan revealed that pantothenamides are phosphorylated but remain trapped in the active site [7]. Given the differential effects of many of our compounds towards *S. aureus* and *S. pyogenes*, it is likely that the modes of action are distinct.

Clearly, additional efforts are required to identify improved inhibitors that may progress to the clinic. So far we have relied on ligand-based design, but preferably we would direct future campaigns to structure-based design. We are currently investigating the mechanism of action of the pantothenamide bioisosteres.

## Supplementary information

Sup table 1

Sup table 1 legend

Sup text 1
