# Supplementary material for: Stable pantothenamide bioisosteres: novel antibiotics for Gram-positive bacteria
Source: J Antibiot (Tokyo). 2019 Jun 6;72(9):682–92. doi: 10.1038/s41429-019-0196-6 (PMC6760626; doi:10.1038/s41429-019-0196-6)
Supplement: Supplementary file 1 — Sup table 1 [file 41429_2019_196_MOESM1_ESM.pdf]

Compound	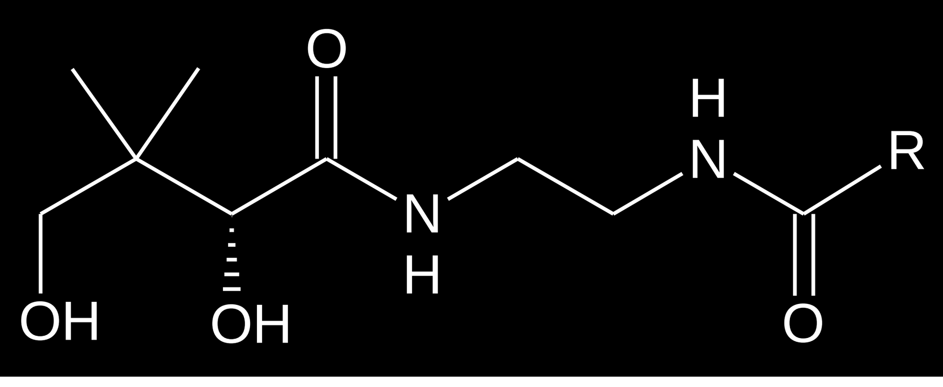	*S.aureus* ATCC6538	*S.epidermidis* ATCC12228	*S.pyogenes* SS91	*E.coli* ATCC25922	*P.aeruginosa* ATCC15692	*M.avium* ATCC700898	*M.abscessus* CIP105536	*M.kansasii* ATCC25221
CXP14.18-027	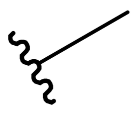	>32	>32	>32	>32	>32	>32	>32	>32
CXP14.18-019	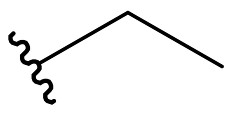	>32	>32	>32	>32	>32	>32	>32	>32
CXP14.18-020	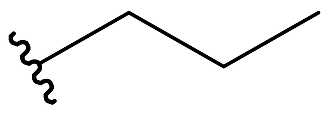	>32	>32	32	>32	>32	>32	>32	>32
CXP14.18-028	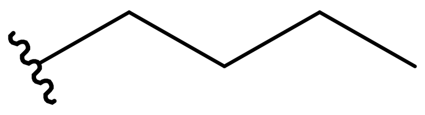	>32	>32	2	>32	>32	>32	>32	>32
CXP18.6-013	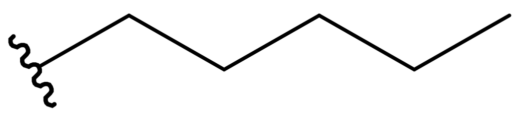	32	16	2	>32	>32	>32	>32	>32
CXP18.6-012	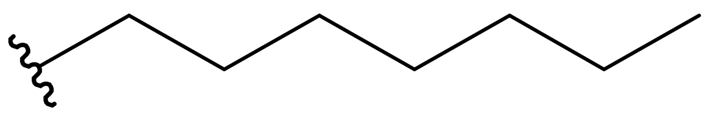	2	8	32	>32	>32	>32	>32	>32
CXP18.6-014	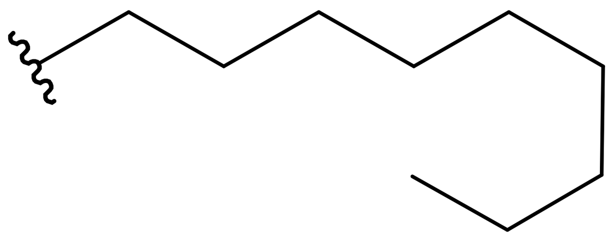	4	2	32	>32	>32	>32	>32	>32
CXP14.18-012	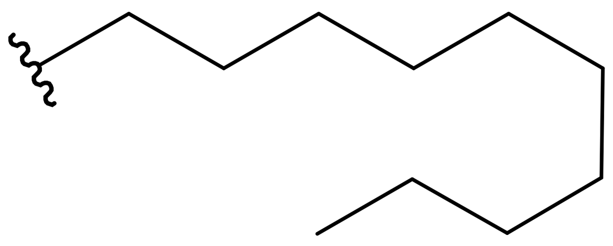	>32	2	16	>32	>32	>32	>32	>32
CXP14.18-013	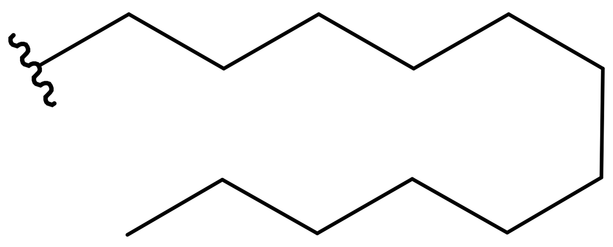	>32	>32	>32	>32	>32	>32	>32	>32
CXP14.18-007	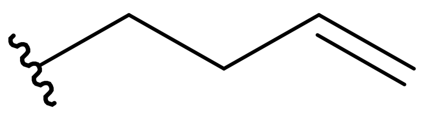	>32	>32	16	>32	>32	>32	>32	>32
CXP14.18-008	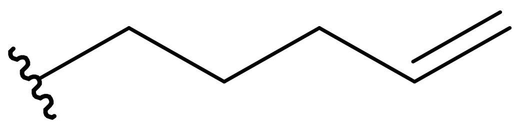	>32	>32	16	>32	>32	>32	>32	>32
CXP14.18-010	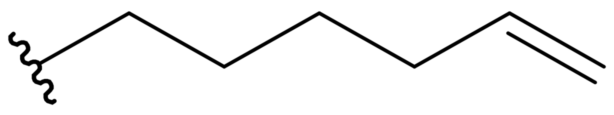	>32	>32	16	>32	>32	>32	>32	>32

Compound	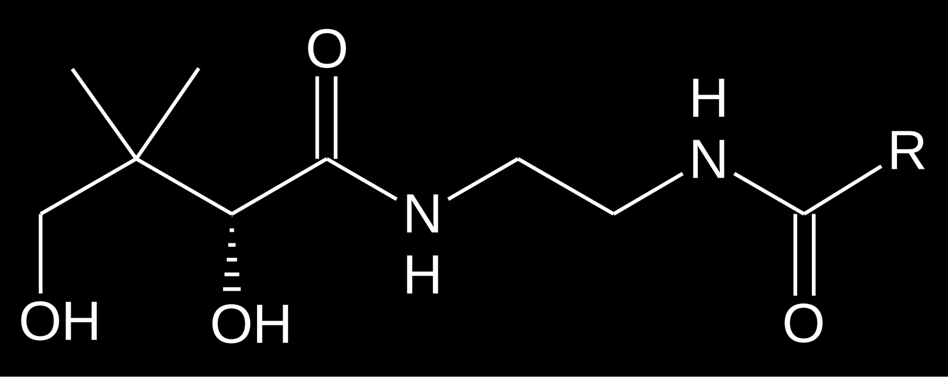	*S.aureus* ATCC6538	*S.epidermidis* ATCC12228	*S.pyogenes* SS91	*E.coli* ATCC25922	*P.aeruginosa* ATCC15692	*M.avium* ATCC700898	*M.abscessus* CIP105536	*M.kansasii* ATCC25221
CXP14.18-011	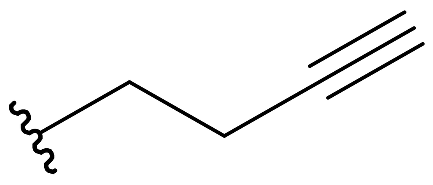	>32	>32	>32	>32	>32	>32	>32	>32
CXP14.18-016	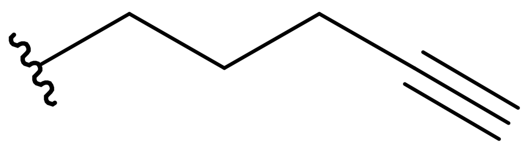	>32	>32	32	>32	>32	>32	>32	>32
CXP14.18-005	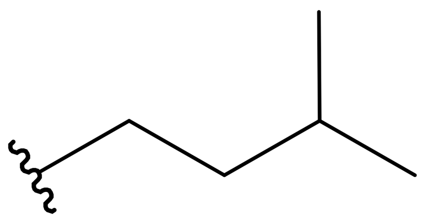	>32	>32	8	>32	>32	>32	>32	>32
CXP14.18-014	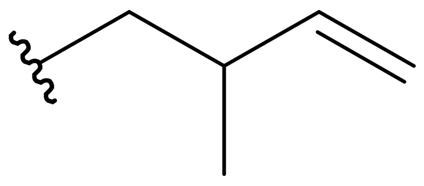	>32	>32	16	>32	>32	>32	>32	>32
CXP14.18-015	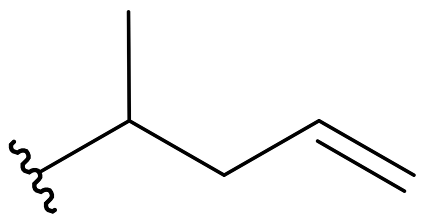	>32	>32	>32	>32	>32	>32	>32	>32
CXP14.26-043	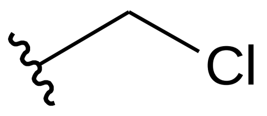	>32	>32	>32	>32	>32	>32	>32	>32
CXP14.26-007	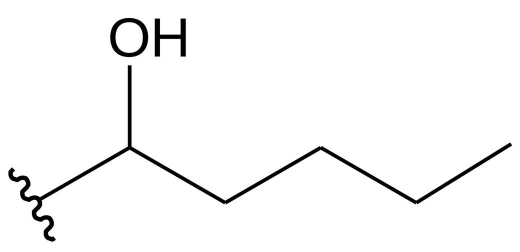	>32	>32	8	>32	>32	>32	>32	>32
CXP14.26-046	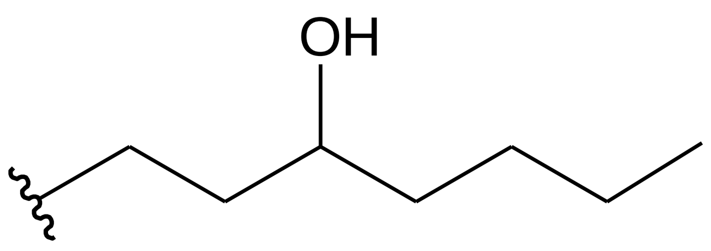	>32	>32	>32	>32	>32	>32	>32	>32
CXP14.18-036	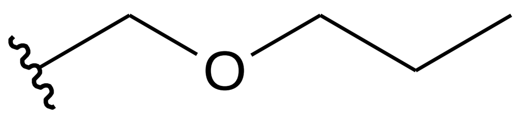	>32	>32	>32	>32	>32	>32	>32	>32
CXP14.18-035	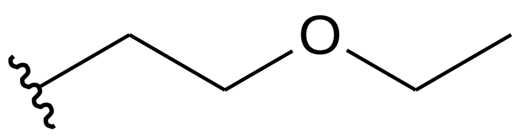	>32	>32	16	>32	>32	>32	>32	>32
CXP14.18-037	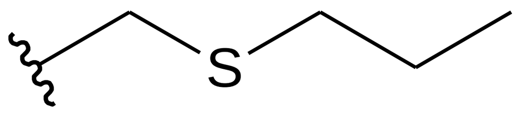	>32	32	4	>32	>32	>32	>32	>32
CXP14.18-038	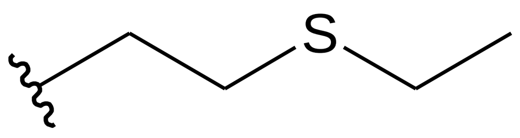	>32	>32	8	>32	>32	>32	>32	>32

Compound	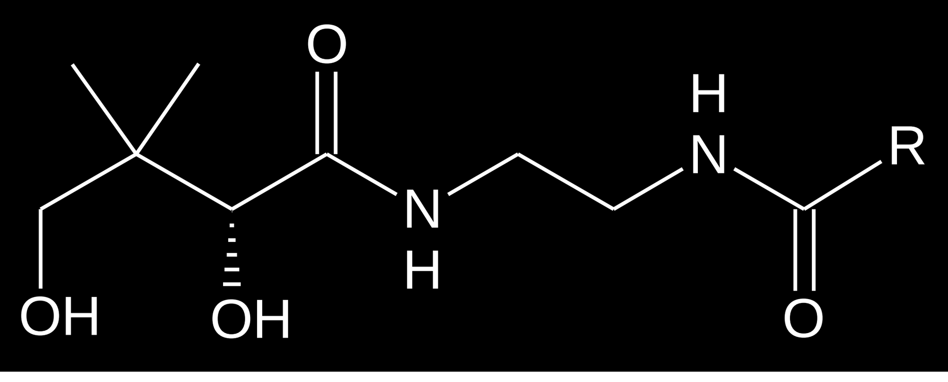	*S.aureus* ATCC6538	*S.epidermidis* ATCC12228	*S.pyogenes* SS91	*E.coli* ATCC25922	*P.aeruginosa* ATCC15692	*M.avium* ATCC700898	*M.abscessus* CIP105536	*M.kansasii* ATCC25221
CXP14.18-034	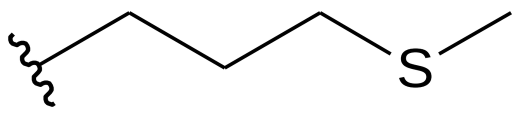	>32	>32	8	>32	>32	>32	>32	>32
CXP14.26-056	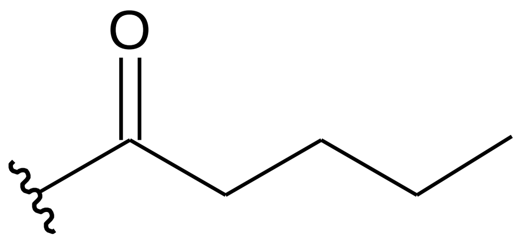	>32	>32	>32	>32	>32	>32	>32	>32
CXP14.26-012	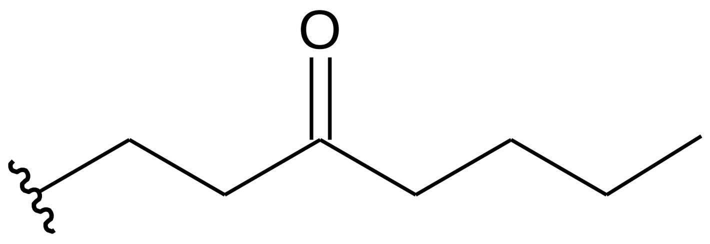	>32	>32	>32	>32	>32	>32	>32	>32
CXP14.26-009	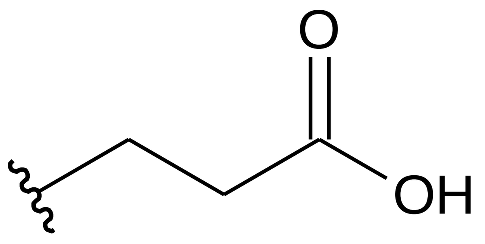	>32	>32	>32	>32	>32	>32	>32	>32
CXP14.26-034	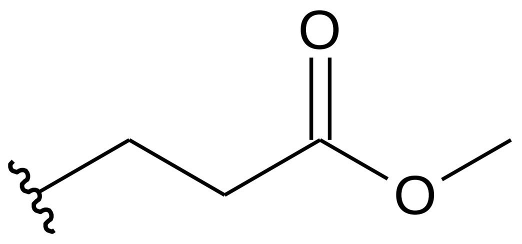	>32	>32	>32	>32	>32	>32	>32	>32
CXP14.26-033	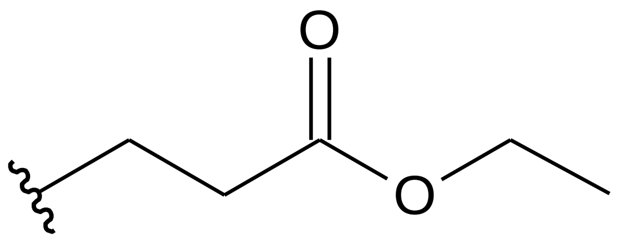	>32	>32	>32	>32	>32	>32	>32	>32
CXP14.26-029	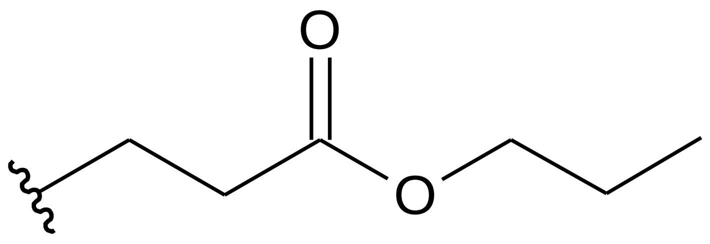	>32	>32	>32	>32	>32	>32	>32	>32
CXP14.26-020	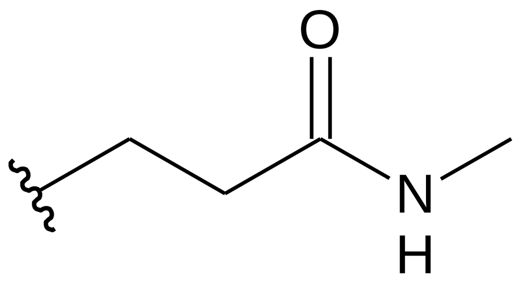	>32	>32	>32	>32	>32	>32	>32	>32
CXP14.26-036	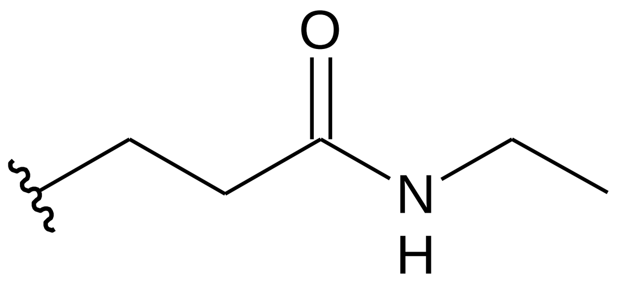	>32	>32	>32	>32	>32	>32	>32	>32
CXP14.26-037	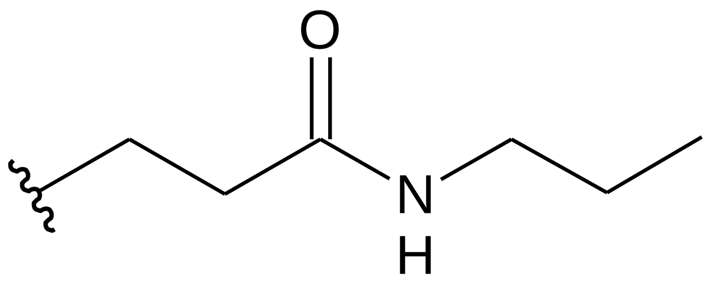	>32	>32	>32	>32	>32	>32	>32	>32
CXP14.26-042	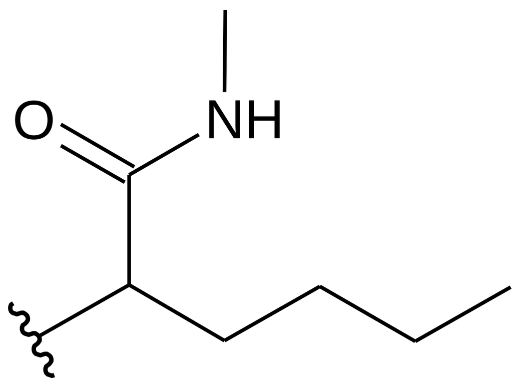	>32	>32	>32	>32	>32	>32	>32	>32
CXP18.6-007	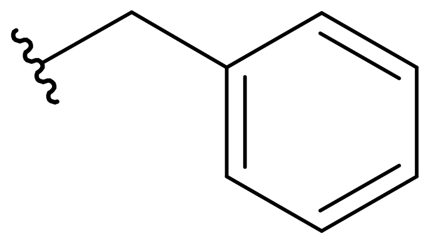	>32	>32	16	>32	>32	>32	>32	>32

Compound	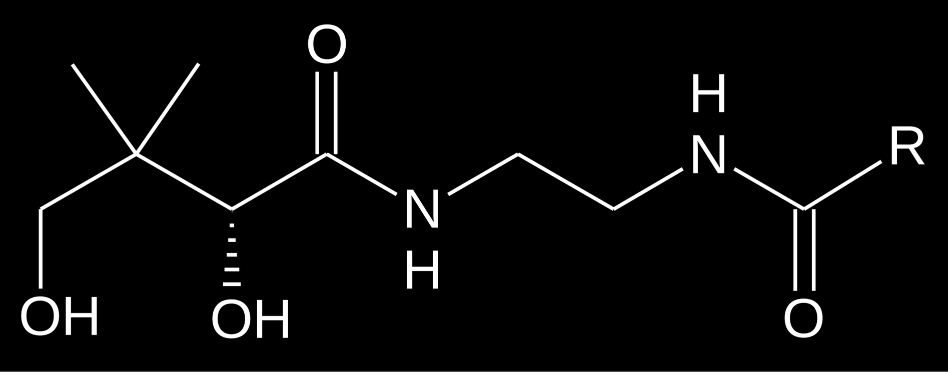	*S.aureus* ATCC6538	*S.epidermidis* ATCC12228	*S.pyogenes* SS91	*E.coli* ATCC25922	*P.aeruginosa* ATCC15692	*M.avium* ATCC700898	*M.abscessus* CIP105536	*M.kansasii* ATCC25221
CXP18.6-006	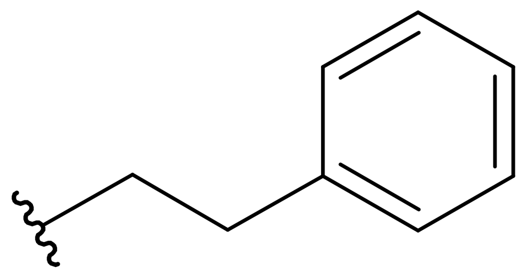	>32	16	>32	>32	>32	>32	>32	>32
CXP18.6-008	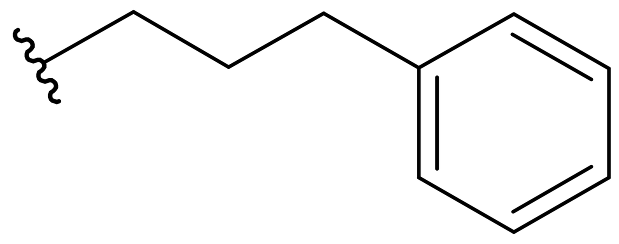	>32	16	>32	>32	>32	>32	>32	>32
CXP14.18-023	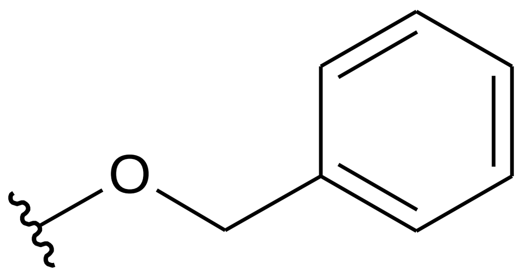	>32	>32	>32	>32	>32	>32	>32	>32
CXP18.6-048	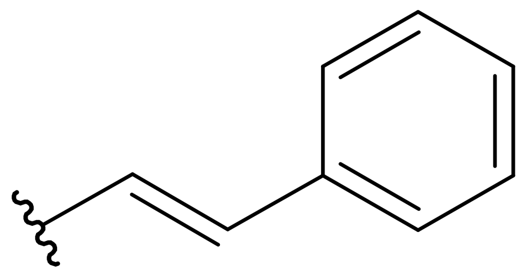	>32	16	>32	>32	>32	>32	>32	>32
CXP18.6-024	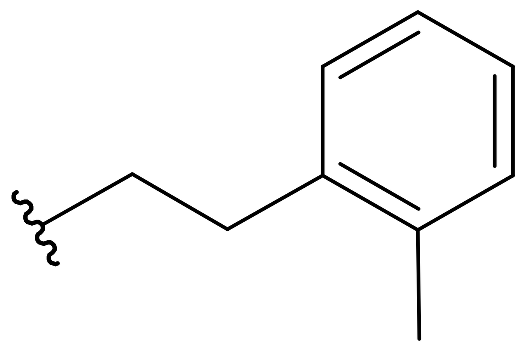	>32	>32	>32	>32	>32	>32	>32	>32
CXP18.6-025	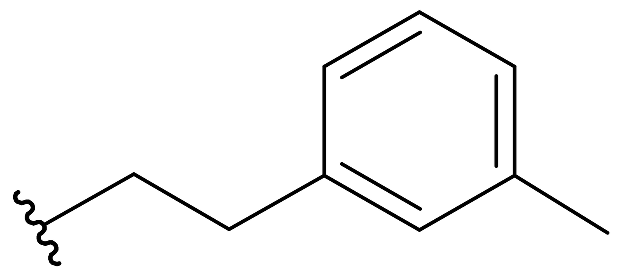	>32	>32	>32	>32	>32	>32	>32	>32
CXP18.6-026	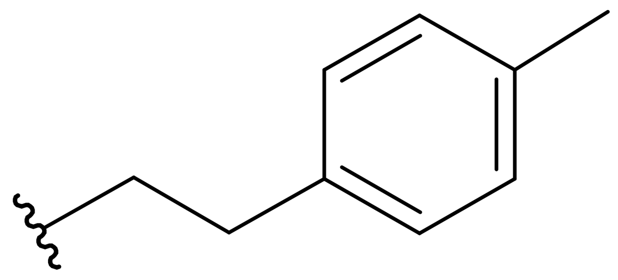	>32	>32	>32	>32	>32	>32	>32	>32
CXP18.6-064	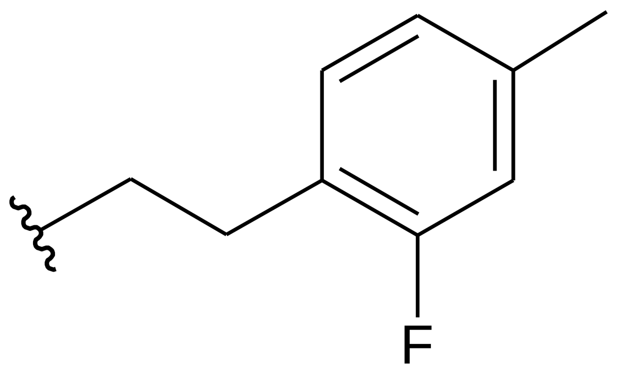	4	1	>32	>32	>32	>32	>32	>32
CXP18.6-017	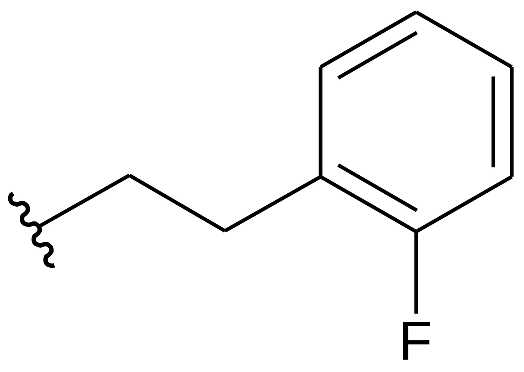	2	2	>32	>32	>32	>32	>32	>32
CXP18.6-018	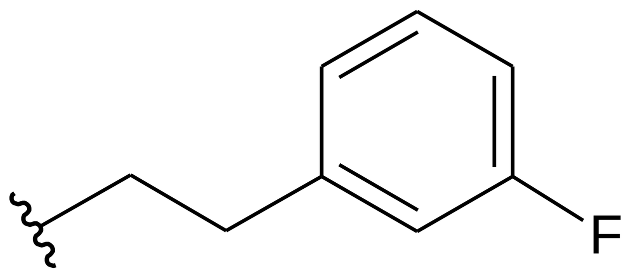	>32	32	16	>32	>32	>32	>32	>32
CXP18.6-019	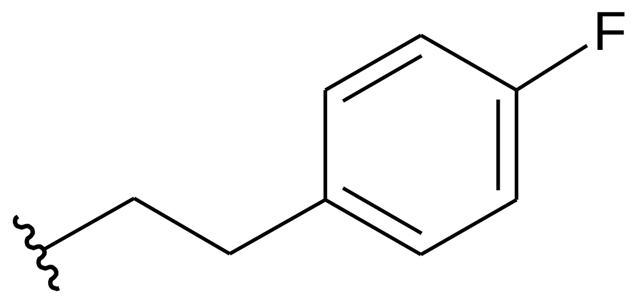	>32	32	>32	>32	>32	>32	>32	>32
CXP18.6-069	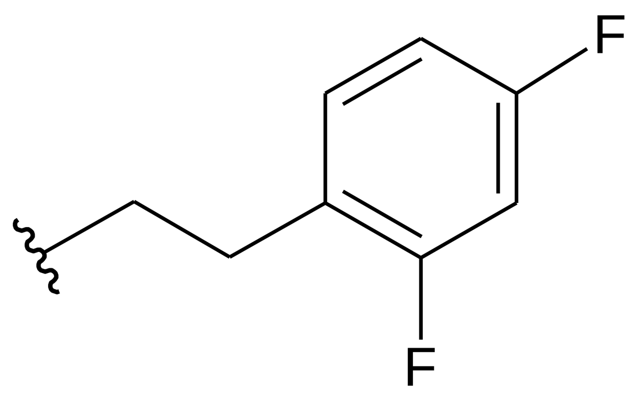	8	1	>32	>32	>32	>32	>32	>32

Compound	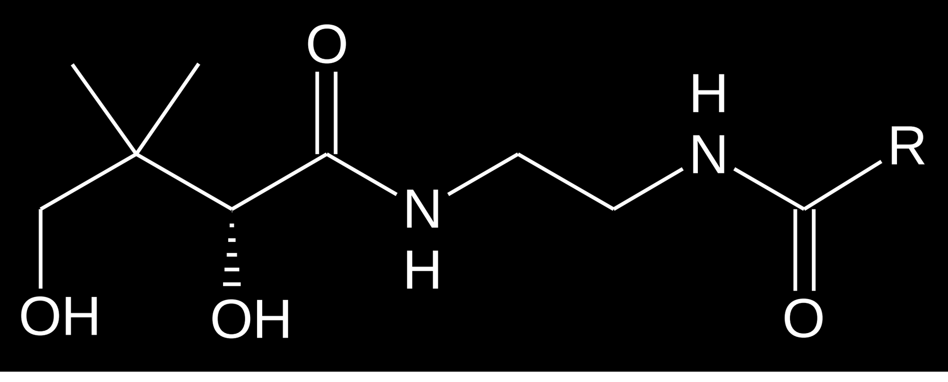	*S.aureus* ATCC6538	*S.epidermidis* ATCC12228	*S.pyogenes* SS91	*E.coli* ATCC25922	*P.aeruginosa* ATCC15692	*M.avium* ATCC700898	*M.abscessus* CIP105536	*M.kansasii* ATCC25221
CXP18.6-038	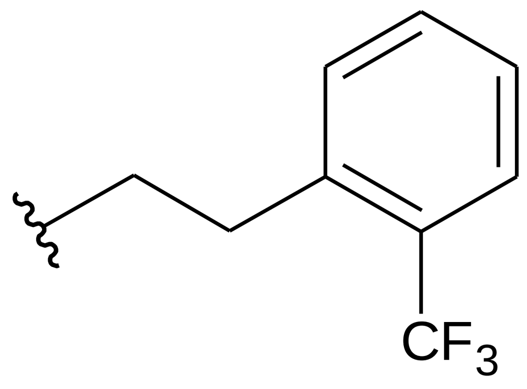	>32	32	>32	>32	>32	>32	>32	>32
CXP18.6-040	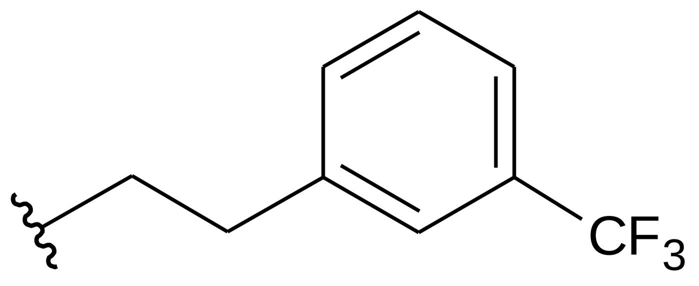	>32	>32	>32	>32	>32	>32	>32	>32
CXP18.6-042	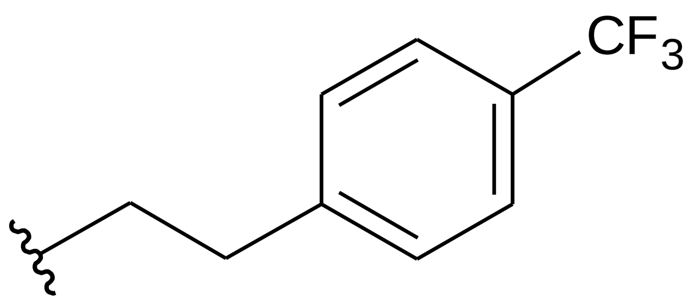	>32	>32	>32	>32	>32	>32	>32	>32
CXP18.6-020	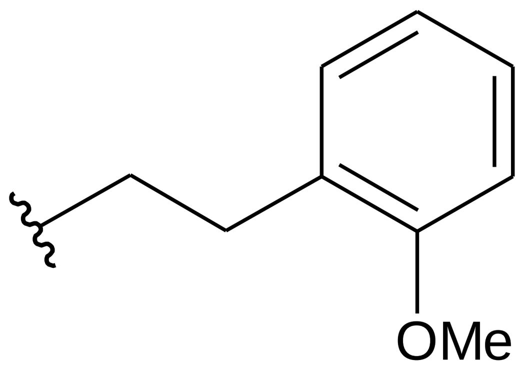	>32	>32	>32	>32	>32	>32	>32	>32
CXP18.6-021	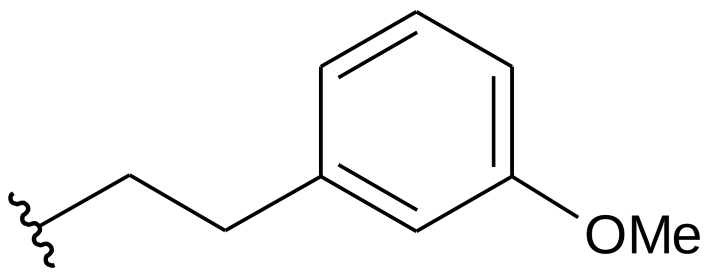	>32	>32	>32	>32	>32	>32	>32	>32
CXP18.6-022	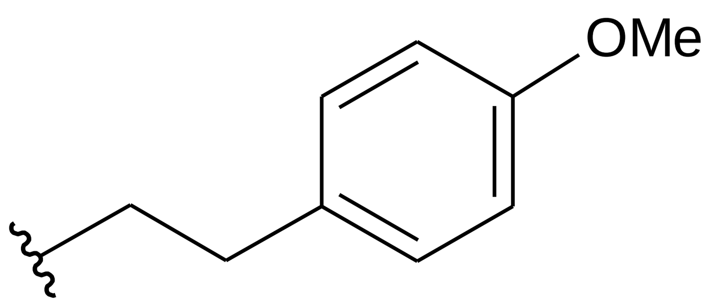	>32	>32	>32	>32	>32	>32	>32	>32
CXP18.6-023	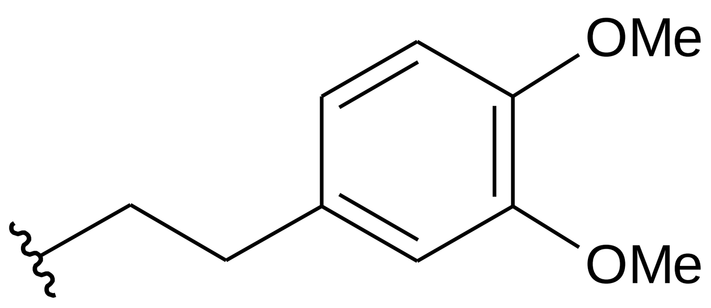	>32	>32	>32	>32	>32	>32	>32	>32
CXP18.6-027	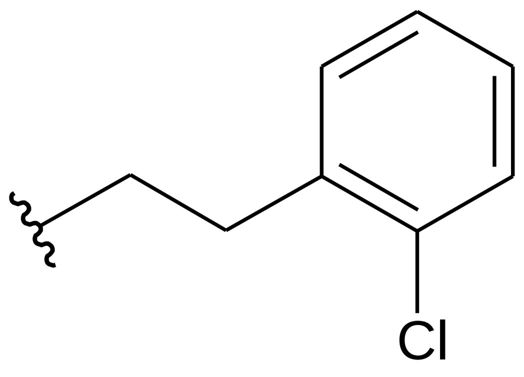	>32	>32	>32	>32	>32	>32	>32	>32
CXP18.6-028	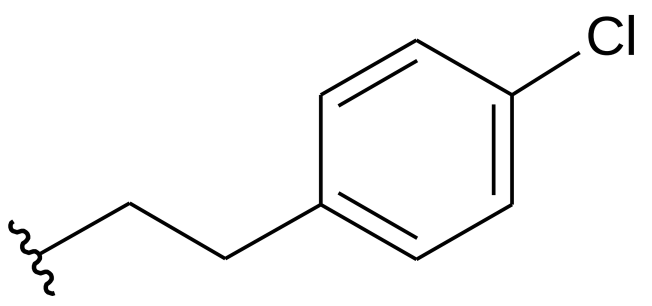	>32	>32	>32	>32	>32	>32	>32	>32
CXP18.6-052	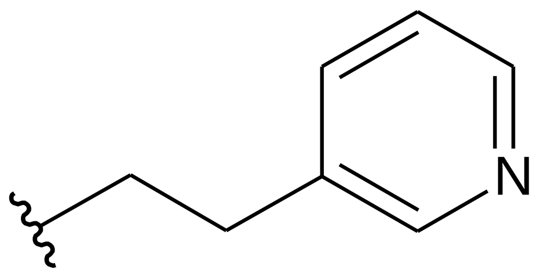	>32	>32	>32	>32	>32	>32	>32	>32
CXP18.6-055	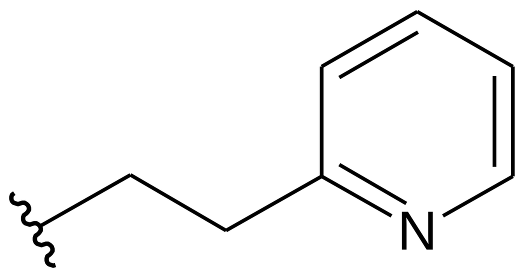	>32	32	>32	>32	>32	>32	>32	>32
CXP18.6-056	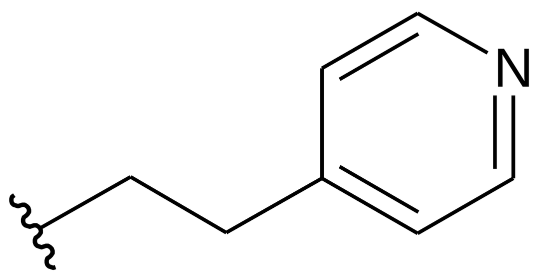	>32	>32	>32	>32	>32	>32	>32	>32

Compound	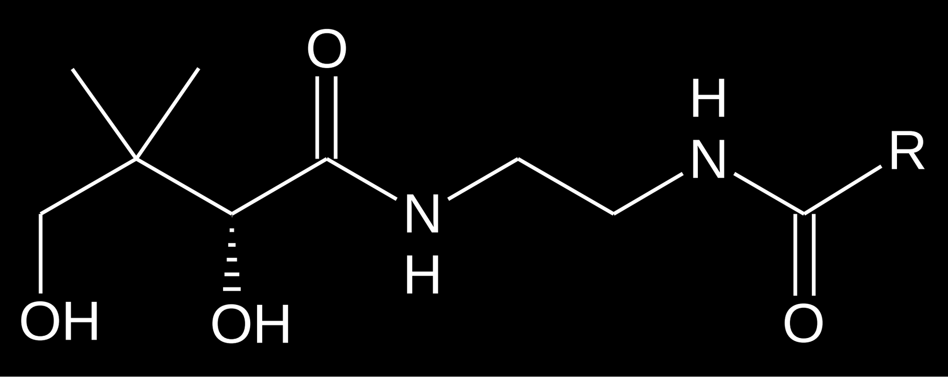	*S.aureus* ATCC6538	*S.epidermidis* ATCC12228	*S.pyogenes* SS91	*E.coli* ATCC25922	*P.aeruginosa* ATCC15692	*M.avium* ATCC700898	*M.abscessus* CIP105536	*M.kansasii* ATCC25221
CXP18.6-090	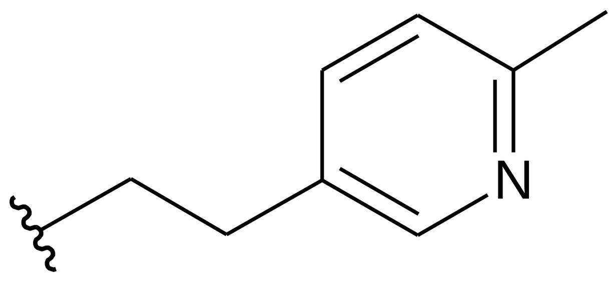	>32	>32	>32	>32	>32	>32	>32	>32
CXP18.6-097	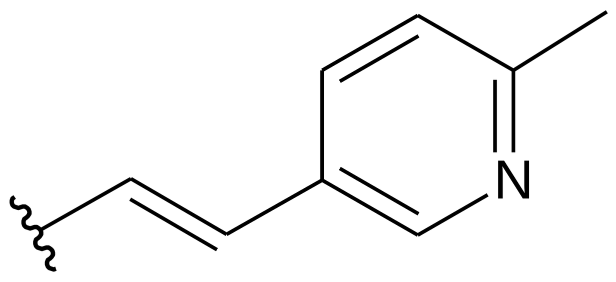	>32	>32	>32	>32	>32	>32	>32	>32
CXP18.6-100	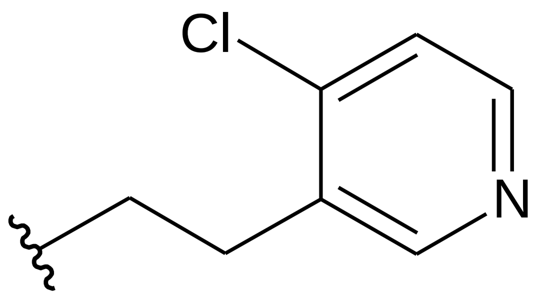	>32	>32	>32	>32	>32	>32	>32	>32
CXP18.6-102	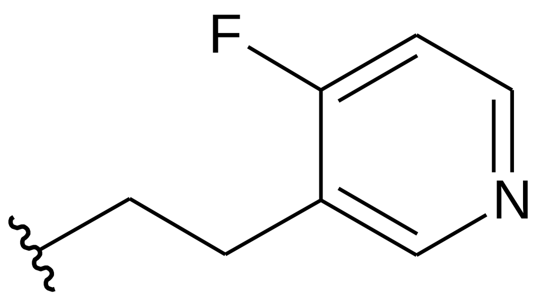	>32	>32	>32	>32	>32	>32	>32	>32
CXP18.6-043	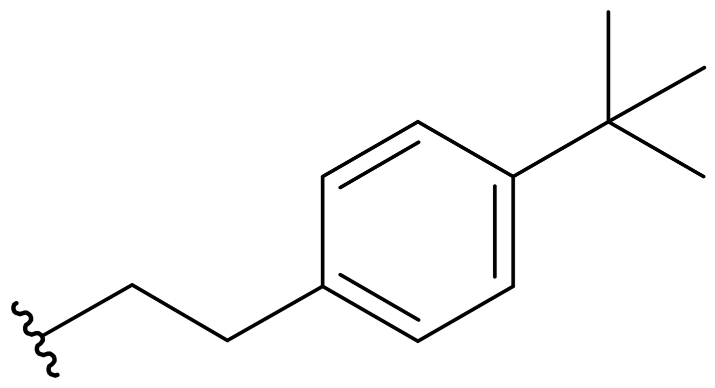	>32	>32	>32	>32	>32	>32	>32	>32
CXP18.6-044	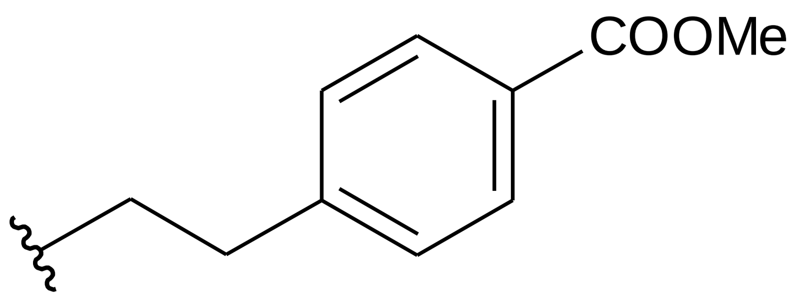	>32	>32	>32	>32	>32	>32	>32	>32
CXP18.6-045	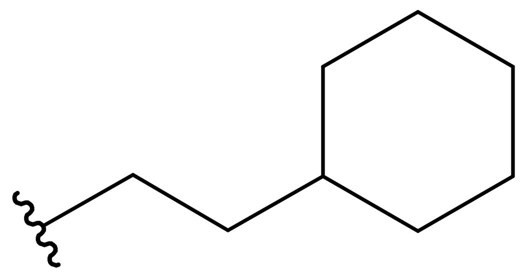	>32	>32	>32	>32	>32	>32	>32	>32
CXP18.6-057	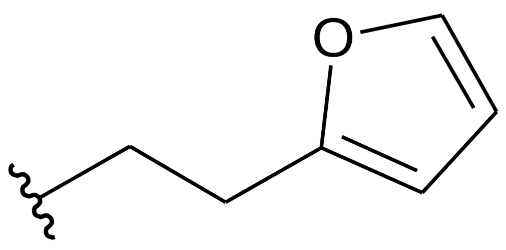	16	8	4	>32	>32	>32	>32	>32
CXP14.26-006	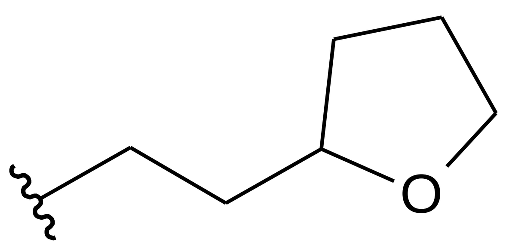	>32	>32	>32	>32	>32	>32	>32	>32
CXP18.6-046	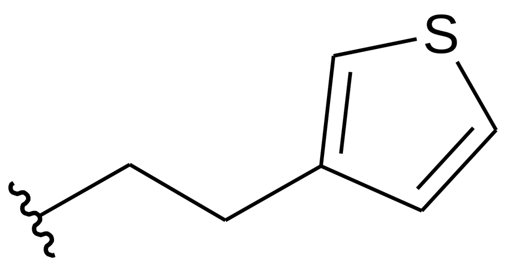	>32	>32	32	>32	>32	>32	>32	>32
CXP18.6-047	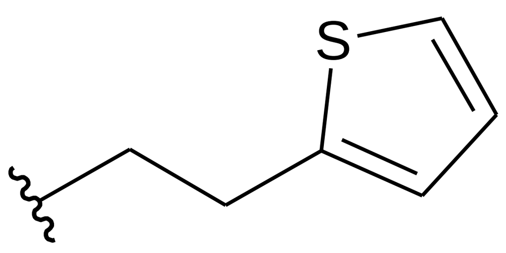	16	16	8	>32	>32	>32	>32	>32
CXP14.18-021	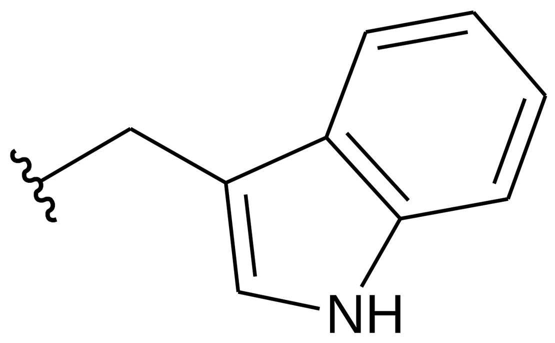	>32	>32	>32	>32	>32	>32	>32	>32
