# Supplementary material for: Stable pantothenamide bioisosteres: novel antibiotics for Gram-positive bacteria
Source: J Antibiot (Tokyo). 2019 Jun 6;72(9):682–92. doi: 10.1038/s41429-019-0196-6 (PMC6760626; doi:10.1038/s41429-019-0196-6)
Supplement: Supplementary file 2 — Sup table 1 legend [file 41429_2019_196_MOESM2_ESM.docx]

Supplementary table 1: MIC of inverted pantothenamide library on different bacteria

See separate file
