# Supplementary material for: Stable pantothenamide bioisosteres: novel antibiotics for Gram-positive bacteria
Source: J Antibiot (Tokyo). 2019 Jun 6;72(9):682–92. doi: 10.1038/s41429-019-0196-6 (PMC6760626; doi:10.1038/s41429-019-0196-6)
Supplement: Supplementary file 3 — Sup text 1 [file 41429_2019_196_MOESM3_ESM.docx]

**General remarks**

Standard syringe techniques were applied for the transfer of air sensitive reagents and dry solvents. Reactions were followed, and Rf values are obtained using thin layer chromatography (TLC) on silica gel-coated plates (Merck 60 F254) with the indicated eluent and compounds were detected with UV-light and/or charring at 150 °C after dipping into a solution of potassium permanganate. Flash column chromatography was carried out using ACROS silica gel (0.035–0.070 mm). ^1^H NMR spectra were recorded at 298 K on Varian 400 (400 MHz) spectrometer in CDCl_3_ or MeOD. Chemical shifts are given in parts per million (ppm) with respect to tetramethylsilane (0.00 ppm), as internal standard for CDCl_3_, or with respect to the solvent residual signal; 3.31 ppm for CD_3_OD. Coupling constants are reported in *J* in Hertz (Hz). LCMS spectra were run on a GraceSmart RP C_18_ 5u column (l x b = 150 x 4.6 mm, particle size 3 µm) with as eluent a MeCN/H_2_O gradient from 50:50 to 95:5 (+ 0.1% TFA). In the Mass Spectrometer (MS), the outcome of the LC was chemically ionized at atmospheric pressure (APCI method) with a limit of detection of 1 m/z. The enantiomeric excess was determined by HPLC (Column: LUX Cellulose-1, Eluent: Hexane/ 2-propanol (85:15% v/v), Flow: 0.5 mL/min, Detection: 254 and 215 nm.

*General procedures*

**A** Grignard alkylation

Under N_2_-conditions, **3** (500 mg, 1.65 mmol) was dissolved in distilled and Na2SO4 dried THF (8 ml) and cooled to 4 °C with an ice bath. 1M Et_2_O solution of the Grignard reagent (5 mL, 5 mmol) was added dropwise over ~1 h to the reaction mixture. The reaction was stirred overnight and monitored by TLC (Heptane : EtOAc = 1 : 3, starting material R_f_ = 0.17, product R_f_ = 0.5). The reaction was diluted with sat. aq. NH_4_Cl (20 ml). After stirring the mixture was diluted with further sat. aq. NH_4_Cl (20 ml), water (5 ml) and EtOAc (40 ml) and stired until most of the solids dissolved. After layer separation the aqueous layer was extracted with EtOAc (40 ml). The combined organic layers were washed with sat. aq. NH_4_Cl (20 ml), water (20 ml) and brine (20 ml), dried over Na_2_SO_4_ and concentrated *in vacuo*. The crude was purified by flash chromatography (DCM / MeOH, from 99 : 1 to 97 : 3)

**B** deprotection from the acetonide clip

To a solution of acetonide protected product (1 equiv.) in MeCN (0.2 M) was added 0.2 M aq. HCl (same volume as MeCN). The reaction was stirred at rt and monitored by TLC or LCMS. Upon completion, the solution was added dropwise to a mixture of sat. aq. NaHCO_3_ (2 ml) and EtOAc (10 ml). The layers were separated and the aqueous layer was extracted with EtOAc (2 × 5 ml).The combined organic layers were dried over Na_2_SO_4_. and the solvents were removed *in vacuo.* The residue was co-evaporated twice with DCM and dried under high vacuum overnight...

**C** 1,2,4-oxadiazole synthesis

To a solution of an amidoxime (1.1 equiv.) in 1,4-dioxane : MeCN = 2 : 1 (0.05M) in a sealed vessel, were added EDCl (1.2 equiv.), DIPEA (1 equiv.) and an acid (1 equiv.). The reaction heated in the MW for 0.25 h to 60° C at 150 W. The vessel was allowed tocool down to rt and heated again in the MW for 0.5 h to 110 °C at 200 W. The reaction mixture was diluted with 5 volumes of EtOAc and washed with sat. aq. NH_4_Cl (2 × 1 volume of reaction solvent), sat. aq. NaHCO_3_ (1 volume of reaction solvent), brine (1 volume of reaction solvent). The organic layer was dried over Na_2_SO_4_ and solvents were removed *in vacuo.* The crude was purified by flash chromatography (Heptane / EtOAc, from 60 : 40 to 40 :60).

**(*R*)-3-(2,4-dihydroxy-3,3-dimethylbutanamido)propanoic acid (1)**

Hemicalcium pantothenate (10 g, 21 mmol) was added to 1M aqueous HCl (50.5 ml, 50.5 mmol) and the suspension was vigorously stirred until the starting material was completely dissolved. NaCl was added till saturation and the suspension was extracted with EtOAc (3 × 100 mL and 3 × 50 mL). The organic layers were collected and dried over Na_2_SO_4_ and solvents were removed *in vacuo*. The product was obtained as a colorless oil (8.5 g, 87%). (M+H)^+^ = 220 m/z

**(*R*)-3-(2,2,5,5-tetramethyl-1,3-dioxane-4-carboxamido)propanoic acid (2)**

**2** (7.6 g, 31 mmol) was dissolved in acetone (100 mL) and cooled on ice to 4 °C. 2-methoxyprop-1-ene (8.9 mL, 93 mmol) and TsOH (0.29 g, 1.5 mmol) were added and after 15 minutes the temperature of the brown solution was raised to rt. After 1.5 h the mixture was diluted with sat. aq. NaHCO_3_ (10 mL). Volatiles were removed *in vacuo* to give a brown oil which was dissolved in an 6% aqueous NaHCO_3_ (150 mL) and extracted with EtOAc (2 × 25 mL). The waterlayer was diluted with EtOAc (50 mL) and the mixture was acidified with sat. aq. KHSO_4_ (~33 mL) until pH 3. After layer separation, the aqueous phase was extracted with EtOAc (50 mL and 20 mL). The organic layers were combined, washed with brine (25 mL), dried over Na_2_SO_4_ and solvents were removed *in vacuo*. The product was obtained as a white powder (6.4 g, 79%).^1^H NMR (CDCl_3_): δ 7.03 (bs, 1H), 4.47 (bs, 2H), 4.11 (s, 1H), 3.70 (d, 1H, *J* = 11.6), 3.55 (m, 2H), 3.28 (d, 1H, *J* = 11.6), 2.63 (t, 2H, *J* = 6.4), 1.46 (s, 3H), 1.43 (s, 3H), 1.04 (s, 3H), 0.98 (s, 3H). (M+H)^+^ = 260 m/z

**(*R*)-*N*-(3-(methoxy(methyl)amino)-3-oxopropyl)-2,2,5,5-tetramethyl-1,3-dioxane-4-carboxamide (3)**

To a cooled (4 °C) solution of 2 (89.5 g, 340 mmol) in DCM (1.15 L) were added subsequently EDC (81.0 g, 425 mmol), N,O-dimethylhydroxylamine.HCl (49.7 g, 509 mmol). The icebath was removed and triethylamine (0.142 L 1020 mmol) was added while the mixture was still cold. After ~10 min DMAP (20.7 g, 170 mmol) was added and the white suspension was stirred at rt and monitored by LCMS. After 4.5 h. the suspension was filtered and the residue was rinsed with CHCl_3_ (200 mL) and DCM (150 mL). The filtrate was diluted with sat. aq. NH_4_Cl (1 L) and water (0.5 L). Under stirring the mixture was acidified with sat. aq. KHSO_4_ until pH 3 (~300 mL). After layer separation the waterlayer was extracted with CHCl_3_ (250 mL). The combined organic layers were washed with 5% aq. citric acid (300 mL), H_2_O (300 mL), sat. aq. NaHCO_3_ (500 mL) and brine (500 mL), dried over Na_2_SO_4_ and solvents were removed *in vacuo*. Yellow oil (110.3 g 100%). ^1^H NMR (CDCl_3_): δ 7.08 (s, 1H), 4.07 (s, 1H), 4.09 (s, 1H), 3.68 (d, 1H, *J* = 11.7), 3.67 (s, 3H), 3.58 (m, 2H), 3.28 (d, 1H, *J* = 11.7), 1.47 (s, 3H), 1.42 (s, 3H), 1.04 (s, 3H), 0.97 (s, 3H). (M+H)^+^ = 303 m/z

**(*R*)-2,2,5,5-tetramethyl-*N*-(3-oxodecyl)-1,3-dioxane-4-carboxamide (4)**

Compound prepared following general procedure **A** using heptylmagnesium chloride. Transparent oil (374 mg, 66%). ^1^H NMR (CDCl_3_): δ 6.93 (s, 1H), 4.05 (s, 1H), 3.67 (d, 1H, *J* = 11.6), 3.50 (m, 2H), 3.27 (d, 1H, *J* = 11.6), 2.67 (t, 2H, *J* = 6.0), 2.39 (t, 2H, *J* = 7.6), 1.56 (m, 2H), 1.46 (s, 3H), 1.41 (s, 3H), 1.26 (m, 8H), 1.04 (s, 3H), 0.95 (s, 3H), 0.88 (t, 3H, *J* = 7.2). (M+H)^+^ = 342 m/z

**(*R*)-2,2,5,5-tetramethyl-*N*-(3-oxoundecyl)-1,3-dioxane-4-carboxamide (5)**

Compound prepared following general procedure **A** using octyllmagnesium bromide. Transparent oil (151 mg, 26%).(M+H)^+^ = 356 m/z

**(*R*)-2,4-dihydroxy-3,3-dimethyl-*N*-(3-oxononyl)butanamide (6; CXP14.19-006)**

Compound prepared following general procedure **B** using **4**. Transparent oil (130 mg, 86%). ^1^H NMR (CDCl_3_): δ 7.12 (s, 1H), 3.99 (s, 1H), 3.51 (m, 5H), 3.01 (bs, 1H), 2.68 (t, 2H, *J* = 6.0), 2.41 (t, 2H, *J* = 7.6), 1.56 (m, 2H), 1.27 (m, 6H), 1.01 (s, 3H), 0.88 (m, 6H). (M+H)^+^ = 288 m/z

**(*R*)-2,4-dihydroxy-3,3-dimethyl-*N*-(3-oxoundecyl)butanamide (7; CXP14.19-005)**

Compound prepared following general procedure **B** using **5**. Transparent oil (109 mg, 82%). ^1^H NMR (CDCl_3_): δ 7.23 (s, 1H), 3.99 (s, 1H), 3.51 (m, 5H), 3.01 (bs, 1H), 2.68 (t, 2H, *J* = 6.0), 2.41 (t, 2H, *J* = 7.2), 1.56 (m, 2H), 1.26 (m, 10H), 1.01 (s, 3H), 0.89 (m, 6H). (M+H)^+^ = 316 m/z

**(*R*)-heptyl 3-(2,2,5,5-tetramethyl-1,3-dioxane-4-carboxamido)propanoate (8)**

To a stirring solution of compound **2** (150 mg, 0.58 mmol) in DCM : THF = 1 : 1 (4 mL) were added EDC (90 mg, 0.58 mmol), DMAP (10 mg, 0.08 mmol) and heptan-1-ol (0.12 mL, 0.87 mmol). The reaction was heated at the MW 0.33 h, 60°C. The mixture was diluted with sat. aq. NH_4_Cl (18 mL), H_2_O (2 mL) and (DCM). After layer separation the waterlayer was extracted with DCM (2 × 15 mL). The organic layers were combined, washed with sat. aq. NH_4_Cl (8 mL), brine (10 mL) and dried over Na_2_SO_4_. Solvents were removed *in vacuo.* The crude was purified by flash chromatography (Heptane : EtOAc = 70 :30). Colorless oil (124 mg, 60%). ^1^H NMR (CDCl_3_): δ 6.96 (bs, 1H), 4.08 (m, 3H), 3.69 (d, 1H, *J* = 11.6), 3.57 (m, 2H), 3.29 (d, 1H, *J* = 12.0), 2.55 (t, 2H, *J* = 6.0), 1.63 (m, 2H), 1.46 (s, 3H), 1.42 (s, 3H), 1.30 (m, 8H), 1.04 (s, 3H), 0.97 (s, 3H), 0.89 (t, 3H, *J* = 6.8). (M+H)^+^ = 358 m/z

**(*R*)-heptyl 3-(2,4-dihydroxy-3,3-dimethylbutanamido)propanoate (9; CXP14.19-107)**

Compound prepared following general procedure **B** using compound **8**. Colorless oil (98 mg, 87%). ^1^H NMR (CDCl_3_): δ 7.17 (bs, 1H), 4.09 (t, 2H *J* = 6.8), 4.02 (s, 1H), 3.53 (m, 5H), 2.99 (bs, 1H), 2.57 (t, 2H, *J* = 6.0), 1.63 (m, 2H), 1.30 (m, 8H), 1.03 (s, 3H), 0.91 (s, 3H), 0.89 (t, 3H, *J* = 7.2). (M+H)^+^ = 318 m/z

**(*R*)-2,4-dihydroxy-3,3-dimethyl-*N*-undecylbutanamide (10; CXP14.19-002)**

D-Pantolactone (250 mg, 1.92 mmol) was dissolved in ethanol (2 mL). Undecan-1-amine (0.33 mL, 1.5 mmol) and DIPEA (0.55 mL, 3.1 mmol) were added. The reaction was brought to 70 °C and left stirring overnight. Subsequently the mixture was diluted with EtOAc (20 mL) and washed with sat. aq. NH_4_Cl (2 × 10 mL), sat. aq. NaHCO_3_ (2 × 10 mL) and brine (2 × 5 mL). The organic layer was dried over Na_2_SO_4_ and solvents were removed *in vacuo*. The crude was purified by flash column chromatography (Heptane / EtOAc, from 70 : 30 to 50 :50). White solid (385 mg, 83%). ^1^H NMR (CDCl_3_): δ 6.71 (s, 1H), 4.03 (d, 1H, *J* = 4.8), 3.52 (m, 2H), 3.45 (d, 1H, *J* = 4.8), 3.27 (m, 2H), 3.14 (t, 1H, *J* = 6.0), 1.52 (m, 2H), 1.27 (m, 12H), 1.04 (s, 3H), 0.91 (s, 3H), 0.88 (t, 3H, *J* = 7.2). (M+H)^+^ = 302 m/z

**n-octanenitrile (11)**

To a solution of NaCN (1.1 g, 17 mmol) and NaI (0.25 g, 1.7 mmol) in DMF (10 mL), was added dropwise 1-bromoheptane (1.3 mL, 8.4 mmol). The temmperature was raised to 85 °C and the reaction was left under vigorous stirring overnight. The mixture was diluted with Et_2_O (30mL) and washed with water (2 × 10 mL), sat. aq. NaHCO_3_ (2 × 10 mL), 10% aqueous NaHSO_3_ (10mL), sat. aq. NH_4_Cl (10mL) and brine.The oganic layer was dried over Na_2_SO_4_ and solvents were removed *in vacuo.* Orange liquid (1.2 g, 75%). ^1^H NMR (CDCl_3_): δ 1.65 (m, 2H), 1.44 (m, 2H), 1.30 (m, 6H), 0.88 (m, 3H).

***N*-hydroxyoctanimidamide (12)**

To a solution of n-octanitrile **11** (600 mg, 3 mmol) in MeOH (10 mL) was added hydroxylamine (0.29 mL, 4.6 mmol) and subsequently K_2_CO_3_ (170 mg, 1.2 mmol). The reaction mixture was brought to reflux monitored by TLC (Heptane : EtOAc = 1 : 1, starting material R_f_ = 0.8, product R_f_ = 0). After 6 h the reaction was cooled to rt and left overnight in the freezer. The day after crystals were formed. The mixture was diluted with MeOH (10 mL), crystals were filtered off and discarded. Solvents were removed from the filtrate *in vacuo* and the residue was dried under high-vacuum overnight. White crystals (313 mg, 61%). ^1^H NMR (CDCl_3_): δ 2.13 (t, 2H, *J* = 7.2), 1.55 (m, 2H), 1.30 (m, 8H), 0.88 (m, 3H). (M+H)^+^ = 159 m/z

**(*R*)-*N*-(2-(3-heptyl-1,2,4-oxadiazol-5-yl)ethyl)-2,2,5,5-tetramethyl-1,3-dioxane-4-carboxamide (13)**

Compound prepared following general procedure **C** using amidoxime **12** and compound **2**. Purification conditions were slightly different (Heptane / EtOAc, from 30 : 70 to 50 : 50) Yellowish oil (90 mg, 35%). (M+H)^+^ = 382 m/z

**(*R*)-*N*-(2-(3-heptyl-1,2,4-oxadiazol-5-yl)ethyl)-2,4-dihydroxy-3,3-dimethylbutanamide (14; CXP14.19-093)**

Compound prepared following general procedure **B** using compound **13**. Transparent oil (36 mg, 44%). ^1^H NMR (CDCl_3_): δ 7.24(s, 1H), 4.02 (d, 1H, *J* = 24.0), 3.70 (m, 2H), 3.58 (bs, 1H), 3.51 (s, 2H), 3.11 (m, 3H), 2.71 (t, 2H, *J* = 7.6), 1.73 (m, 2H), 1.29 (m, 8H), 1.02 (s, 3H), 0.94 (s, 3H), 0.89 (t, 3H, *J* = 6.4). (M+H)^+^ = 342 m/z

**(*R*)-*N*-(2-cyanoethyl)-2,4-dihydroxy-3,3-dimethylbutanamide (15)**

A solution of 3-aminopropanenitrile (0.5 mL, 7 mmol) and (D)-pantolactone (1.4 g, 10 mmol) and DIPEA (0.24 mL, 1.4 mmol) in EtOH (7 mL) was stirred at 55 °C fo 5 hours. The reaction was monitored by LCMS, and the conversion was assessed to not overcome 55%. The mixture was cooled to rt and diluted with EtOAc (35 mL), sat. aq. NH_4_Cl (10 mL) and H_2_O (0.5 mL). After layer separation the aqueous layers was extracted with EtOAc (3 × 25 mL) and the combined organic layers were washed with sat. aq. NH_4_Cl (2 × 5 mL), brine (5 mL) and dried over Na_2_SO_4_. Solvents were removed *in vacuo* to afford a slightly yellow oil. Stored in the freezer the oil becomes a white solid. The water layers were collected and saturated with NaCl to yield further 400 mg of yellow oil, which was combined to the previous crude and purified by flash chromatography. (EtOAc / MeOH, from 95 : 5 to 90 : 10). Colorless oil (500 mg, 32%). ^1^H NMR (CDCl_3_): δ 7.30 (bs, 1H), 4.08 (s, 1H), 3.56 (m, 4H), 2.66 (m, 2H), 1.06 (s, 3H), 0.97 (s, 3H). (M+H)^+^ = 201 m/z

**(*R*)-*N*-(2-cyanoethyl)-2,2,5,5-tetramethyl-1,3-dioxane-4-carboxamide (16)**

**15** (500 mg, 2.2 mmol) was dissolved in acetone (11 mL) and cooled on ice to 4 °C. 2-methoxyprop-1-ene (0.64 mL, 6.6 mmol) and TsOH (20 mg, 0.1 mmol) were added and after 0.25 h the temperature of the solution was raised to rt. TLC (EOAc : MeOH = 95 : 5, R_f_ SM = 0.36, product R_f_ = 0.76) revealed the reaction reached completion in 1 h. EtOAc (20 mL) was added to the reaction mixture. The organic layer was washed with sat. aq. NH_4_Cl (5mL), sat. aq. NaHCO_3_ (5 mL × 2) and brine (5 mL), dried over Na_2_SO_4_ and solvents removed *in vacuo*. White powder (475 mg, 89%). (M+H)^+^ = 241 m/z

**(*R*)-*N*-(3-(hydroxyamino)-3-iminopropyl)-2,2,5,5-tetramethyl-1,3-dioxane-4-carboxamide (17)**

To a solution of **16** (410 mg, 1.7 mmol) in MeOH (9 ml) is added 50% aqueous hydroxylamine (0.25 ml, 4.5 mmol). The reaction mixture is brought to reflux for 24 h and monitored by LCMS and TLC (EtOAc : MeOH = 95 : 5, starting material R_f_ = 0.78, product R_f_ = 0.27). After cooling to rt, volatiles were removed *in vacuo*. Transparent oil (344 mg, 74%). ^1^H NMR (CDCl_3_): δ 6.91 (bs, 1H), 4.47 (bs, 2H), 4.09 (s, 1H), 3.70 (d, 1H, *J* = 11.6), 3.47 (m, 2H), 3.28 (d, 1H, *J* = 12.0), 2.37 (t, 2H, *J* = 6.8), 1.47 (s, 3H), 1.43 (s, 3H), 1.04 (s, 3H), 0.99 (s, 3H). (M+H)^+^ = 274 m/z

**(*R*)-*N*-(2-(5-heptyl-1,2,4-oxadiazol-3-yl)ethyl)-2,2,5,5-tetramethyl-1,3-dioxane-4-carboxamide (18)**

Compound prepared following general procedure **C** using amidoxime **17** and octanoic acid. Transparent oil (120 mg, 99%). ^1^H NMR (CDCl_3_): δ 7.07(s, 1H), 4.09 (s, 1H), 3.68 (m, 3H), 3.27 (d, 1H, *J* = 12.0), 2.95 (t, 2H, *J* = 6.4), 2.86 (t, 2H, *J* = 7.6), 1.81 (m, 2H), 1.46 (s, 3H), 1.43 (s, 3H), 1.30 (m, 8H), 1.04 (s, 3H), 0.94 (s, 3H), 0.88 (m, 3H). (M+H)^+^ = 382 m/z

**(*R*)-*N*-(2-(5-heptyl-1,2,4-oxadiazol-3-yl)ethyl)-2,4-dihydroxy-3,3-dimethylbutanamide (19; CXP14.19-012)**

Compound prepared following general procedure **B** using compound **18**. Crude purified by flash chromatography (Heptane / EtOAc / MeOH, from 90 : 10 : 0 to 0 : 95 : 5)Transparent oil (45 mg, 45%). ^1^H NMR (CDCl_3_): δ 7.16(s, 1H), 4.01 (s, 1H), 3.70 (m, 2H), 3.51 (m, 2H), 2.97 (t, 2H, *J* = 6.0), 2.87 (t, 2H, *J* = 7.6), 1.80 (m, 2H), 1.33 (m, 8H), 1.03 (s, 3H), 0.94 (s, 3H), 0.88 (m, 3H). (M+H)^+^ = 342 m/z

**Methyl octanoate (20)**

To ice-bath cooled MeOH (20 mL) was slowly added sulfurous dichloride (1.3 mL, 17 mmol). After the addition octanoic acid (1.1 mL, 7.0 mmol) was added and the solution was brought to rt and left stirring overnight. The reaction mixture was diluted with sat. aq. NaHCO_3_ (50 mL) and extracted with DCM (3 × 20 mL). The combined organic layers were washed with further 20 mL of brine, dried over Na_2_SO_4_ and solvents were removed *in vacuo*. Transparent liquid (1.1 g, 93%). ^1^H NMR (CDCl_3_): δ 2.30 (t, 2H, *J* = 8.0), 1.62 (m, 2H), 1.29 (m, 8H), 0.88 (m, 3H).

**Octanehydrazide (21)**

To a solution of **20** (1.1 g, 7.0 mmol) in MeOH (5 mL) 50% aqueous hydrazine (1 mL, 20 mmol) was added and the reaction mixture was brought to reflux for 2 hours. After cooling with an ice-bath, the formed crystals were filtered and left on high vacuum overnight. White crystals (870 mg, 79%). ^1^H NMR (CDCl_3_): δ 2.15 (t, 2H, *J* =7.6), 1.64 (m, 2H), 1.29 (m, 8H), 0.88 (m, 3H). (M+H)^+^ = 159 m/z

**(*R*)-*N*-(3-(2-octanoylhydrazinyl)-3-oxopropyl)-2,2,5,5-tetramethyl-1,3-dioxane-4-carboxamide (22)**

To a solution of compound **2** (0.87 g, 5.5 mmol) in MeCN : H_2_O = 9 : 1 (15mL) HOBt (1.3 g, 8.3 mmol) was added and left stirring 10 minutes at rt. Compound **21** (1.9 g, 7.2 mmol) and EDC (1.6 g, 8.3 mmol) were added and the reaction was left stirring at rt for 3h. The mixture was diluted with EtOAc (15 mL) and washed with sat. aq. NH_4_Cl (2 × 10 mL), sat. aq. NaHCO_3_ (2 × 10 mL) and brine (2 × 10 mL). The organic layer was dried over Na_2_SO_4_ and solvents were removed *in vacuo.* Yellowish solid (1.6 g, 61%). ^1^H NMR (CDCl_3_): δ 8.84 (bs, 1H), 8.29 (bs, 1H), 7.08(s, 1H), 4.09 (s, 1H), 3.61 (d, 1H, *J* = 11.6), 3.58 (m, 2H), 3.27 (d, 1H, *J* = 11.6), 2.54 (t, 2H, *J* = 6.0), 2.25 (t, 2H, *J* = 7.2), 1.65 (m, 2H), 1.46 (s, 3H), 1.42 (s, 3H), 1.30 (m, 8H), 1.03 (s, 3H), 0.97 (s, 3H), 0.88 (m, 3H). (M+H)^+^ = 400 m/z.

**(*R*)-*N*-(2-(5-heptyl-1,3,4-oxadiazol-2-yl)ethyl)-2,2,5,5-tetramethyl-1,3-dioxane-4-carboxamide (23)**

To a solution of compound **22** (250 mg, 0.60 mmol) in DCM (3.2 mL), TEA (0.26 mL, 1.8 mmol) and tosyl chloride (370 mg, 1.9 mmol) were added and the reaction was stirred at rt for 1h. The mixture was diluted with sat. aq. NaHCO_3_ (10 mL) and DCM (10 mL). After layer separation the aqueous layers was extracted with DCM (3 × 10 mL). The organic layers were collected and washed with sat. aq. NH_4_Cl (10 mL) and brine (10 mL). The organic layer was dried over Na_2_SO_4_ and solvent was removed *in vacuo.* The crude was purified by flash chromatography (Heptane / EtOAc, from 30 : 70 to 10 : 90). Transparent oil (127 mg, 53%). ^1^H NMR (CDCl_3_): δ 7.04(bs, 1H), 4.08 (s, 1H), 3.71 (m, 3H), 3.27 (d, 1H, *J* = 11.6), 3.05 (t, 2H, J = 6.8), 2.81 (t, 2H, *J* = 7.6), 1.77 (m, 2H), 1.46 (s, 3H), 1.42 (s, 3H), 1.32 (m, 8H), 1.03 (s, 3H), 0.93 (s, 3H), 0.88 (m, 3H). (M+H)^+^ = 381 m/z

**(*R*)-*N*-(2-(5-heptyl-1,3,4-oxadiazol-2-yl)ethyl)-2,4-dihydroxy-3,3-dimethylbutanamide (24; CXP14.19-027)**

Compound prepared following general procedure **B** using compound **23**. Colorless oil (108 mg, 95%). ^1^H NMR (CDCl_3_): δ 7.38(m, 1H), 4.12 (d, 1H, *J* = 4.8), 4.01 (d, 1H, *J* = 4.0), 3.71 (m, 2H), 3.49 (m, 3H), 3.06 (t, 2H, *J* = 6.8), 2.81 (t, 2H, *J* = 7.6), 1.76 (m, 2H), 1.34 (m, 8H), 1.00 (s, 3H), 0.93 (s, 3H), 0.89 (m, 3H). (M+H)^+^ = 342 m/z

**Tert-butyl (3-(2-octanoylhydrazinyl)-3-oxopropyl)carbamate (25)**

To a solution of *N*-Boc-*beta*-Ala (530 mg, 2.8 mmol) in MeCN (20 mL), HOBt (400 mg, 2.6 mmol), EDCI (490 g, 2.6 mmol) and compound **21** (370 mg, 2.3 mmol) were added and the reaction was left stirring at rt for 1 h. The mixture was diluted with EtOAc (15 mL) and washed with sat. aq.NH_4_Cl (2 × 6 mL), sat. aq. NaHCO_3_ (2 × 6 mL) and brine (6 mL). The organic layer was dried over Na_2_SO_4_ and solvents were removed *in vacuo.* White solid (550 mg, 80%). ^1^H NMR (CDCl_3_): δ 8.50 (bs, 1H), 8.21 (bs, 1H), 5.20 (bs, 1H), 3.43 (m, 2H), 2.49 (m, 2H), 2.26 (t, 2H, *J* = 7.6), 1.66 (m, 2H), 1.43 (s, 9H), 1.29 (m, 8H),0.88 (m, 3H). (M+H)^+^ = 330 m/z

**tert-butyl (2-(5-heptyl-1,3,4-thiadiazol-2-yl)ethyl)carbamate (26)**

Lawesson's reagent (150 mg, 0.37 mmol) was dissolved in THF (4 mL) in a sealed tube. Compound **25** (130 mg, 0.12 mmol) and K_2_CO_3_ (80 mg, 0.6 mmol) were added and the reaction was heated in the MW for 0.3 h to 55 °C at 75W. The mixture was diluted with sat. aq. NaHCO_3_ (8 mL) and extracted with EtOAc (3 × 10 mL). The organic layers were combined, washed with brine (8mL), dried over Na_2_SO_4_ and solvents were removed *in vacuo*. The crude was purified by flash chromatography (Heptane / EtOAc, from 50 : 50 to 30 :70)Yellowish solid (100 mg, 52%). ^1^H NMR (CDCl_3_): δ 5.13 (bs, 1H), 3.60 (q, 2H. *J* = 6.0), 3.25 (t, 2H, *J* = 6.4), 3.07 (t, 2H, *J* = 7.6), 1.78 (m, 2H), 1.43 (m, 9H), 1.34 (m, 8H), 0.89 (m, 3H). (M+H)^+^ = 328 m/z

**2-(5-heptyl-1,3,4-thiadiazol-2-yl)ethanamine hydrochloride (27)**

Through an ice bath cooled solution of **26** (190 mg, 0.50 mmol) in EtOAc (10 mL) was bubbled freshly prepared HCl gas from CaCl_2_ (9 g) and concentrated aqueous HCl (10 mL). The mixture was brought to rt and left stirring for 1 h. Subsequently it was gently poured on stirring Et_2_O (30 mL) and the clear solution was left stirring at rt. After ~0.5 h the solution was cooled without stirring in an ice-bath. The white precipitate was filtered and rinsed with Et_2_O (2 x 10 mL). White solid (140 mg, 88%). ^1^H NMR (CD_3_OD): δ 3.43 (m, 4H), 3.09 (t, 2H. *J* = 7.6), 1.76 (qn, 2H, *J* = 7.6), 1.38(m, 8H), 0.86 (m, 3H). (M+H)^+^ = 228 m/z

**(*R*)-*N*-(2-(5-heptyl-1,3,4-thiadiazol-2-yl)ethyl)-2,4-dihydroxy-3,3-dimethylbutanamide (28; CXP14.19-041)**

**27** (140 mg, 0.5 mmol) was dissolved in EtOH (0.5 mL). D-Pantolactone (210 mg, 1.6 mmol) and DIPEA (0.48 mL, 2.7 mmol) were subsequently added. The reaction was brought to 70 °C and left stirring overnight. The mixture was diluted with EtOAc (20 mL), sat. aq. NH_4_Cl (2 × 10 mL), sat. aq. NaHCO_3_ (2 × 10 mL) and brine (2 × 5 mL). The organic layer was dried over Na_2_SO_4_ and solvents removed *in vacuo*. The crude was purified by flash chromatography (Heptane / EtOAc, from 70 : 30 to 50 : 50). Yellowish solid (61 mg, 32%). ^1^H NMR (CDCl_3_): δ 7.34(bs, 1H), 4.00 (dd, 2H, *J* = 5.2, 20.2), 3.77 (m, 2H), 3.48 (m, 3H), 3.28 (t, 2H, *J* = 6.0), 3.06 (t, 2H, J = 7.6), 1.77 (qn, 2H, *J* = 6.4),, 1.30 (m, 8H), 1.00 (s, 3H), 0.93 (s, 3H), 0.88 (m, 3H). (M+H)^+^ = 358 m/z

**Ethyl octanimidate hydrochloride (29)**

Acetyl chloride (2.6 mL, 36 mmol) was added dropwise to a stirred solution of n-octanitrile **11** (600 µL, 4.5 mmol) in EtOH (2.5 mL, 54 mmol). The reaction flask was sealed tightly and the stirring was continued at rt overnight. N_2_ was bubbled through the mixture for 0.5 h and the volatiles were removed *in vacuo*. Yellowish waxy solid (500 mg, 53%). (M+H)^+^ = 172 m/z

**methyl 3-(((benzyloxy)carbonyl)amino)propanoate (30)**

To ice-bath cooled MeOH (20 mL) was slowly added SOCl_2_ (1.3 mL, 17 mmol). Cbz-β-Alanine (1.0 g, 4.5 mmol) was added, the solution was brought to rt and left stirring overnight. The mixture was diluted with sat. aq. NaHCO_3_ (50 mL) and extracted with DCM (3 × 20 mL). The combined organic layers were washed with further 20 mL of brine and dried over Na_2_SO_4_. Solvents were removed *in vacuo*. Colorless oil (1.1 g, 100%).

**benzyl (3-hydrazinyl-3-oxopropyl)carbamate (31)**

To a solution of compound **30** (1.1 g, 5.2 mmol) in MeOH (5 mL) 50% aqueous hydrazine (1.0 mL, 21 mmol) was added and the reaction mixture was brought to reflux for 3 hours. After cooling with an ice-bath, the formed crystals were filtered and left on high vacuum overnight. White crystals (1.1 g, 100%). ^1^H NMR (CDCl_3_): δ 7.35 (m, 5H), 6.98 (bs, 1H), 5.41 (bs, 1H), 5.09 (s, 2H), 3.88 (bs, 2H), 3.50 (q, 2H, *J* = 6.4), 2.39 (t, 2H, *J* = 6.0). (M+H)^+^ = 238 m/z

**benzyl (2-(5-heptyl-4H-1,2,4-triazol-3-yl)ethyl)carbamate (32)**

A solution of hydrazide **31** (300 mg, 1.3 mmol) and DIPEA (0.50 mL, 2.8 mmol) in DMF (2 mL) were added to heptyl-imidate **29** (290 mg, 1.4 mmol) in a MW sealed vessel, and the reaction was stirred at rt for 1 h. The vessel was heated in the MW for 0.3 h to 60 °C at 150W. The mixture was diluted with EtOAc (20mL), washed with sat. aq. NH_4_Cl (2 × 5 mL) and brine (2 × 4 mL). The organic layer was dried over Na_2_SO_4_ and solvents were removed *in vacuo.* The crude was purified by flash chromatography (Heptane / EtOAc / MeOH, from 10 : 90 : 0 to 0 : 90 : 10). White solid (90 mg, 21%).

**2-(5-heptyl-4H-1,2,4-triazol-3-yl)ethanamine hydrochloride (33)**

Compound **32** (90 mg, 0.3 mmol) was dissolved in 2-propanol (2.6 mL). Pd/C (14 mg, 0.013 mmol) was added and H_2_ (0.5 mg, 0.3 mmol) was bubbled through the solution. The reaction mixture was stirred at rt. for 1h. The mixture was diluted with 1M aq.HCl (0.26 mL, 0.26 mmol) and filtered through celite. The filtrate was diluted with Et_2_O (20 mL) and formed crystals were separated by decantation. The collected solid was re-crystallized from Et_2_O (15 mL). White solid (60 mg, 100%). (M+H)^+^ = 211 m/z

**(*R*)-*N*-(2-(5-heptyl-4H-1,2,4-triazol-3-yl)ethyl)-2,4-dihydroxy-3,3-dimethylbutanamide (32; CXP14.19-094)**

**33** (60 mg, 0.2 mmol) was dissolved in EtOH (0.3 mL). D-Pantolactone (130 mg, 1.0 mmol) and DIPEA (0.3 mL, 1.7 mmol) were subsequently added. The reaction was brought to 70 °C and left stirring overnight. The mixture was diluted with EtOAc (20 mL), sat. aq. NH_4_Cl (2 × 10 mL), sat. aq. NaHCO_3_ (2 × 10 mL) and brine (2 × 5 mL). The organic layer was dried over Na_2_SO_4_ and solvents were removed *in vacuo*. The crude was purified by flash chromatography (EtOAc / MeOH, from 100 : 0 to 85 : 15). Colorless oil (50 mg, 70%). ^1^H NMR (CDCl_3_): δ 7.35 (bs, 1H), 4.59 (bs, 1H), 3.98 (s, 1H), 3.66 (m, 2H), 3.48 (dd, 2H, *J* = 11.2, 26.0), 2.96 (t, 2H, *J* = 6.0), 2.72 (t, 2H, J = 8.0), 1.72 (m, 2H), 1.31 (m, 8H), 1.02 (s, 3H), 0.96 (s, 3H), 0.87 (t, 3H. *J* = 6.8). (M+H)^+^ = 341 m/z

**2-octyloxirane (35)**

non-1-ene (2.4 mL, 14 mmol) was dissolved in DCM (60 mL) and cooled on ice to 4 °C. 3-chlorobenzoperoxoic acid (7.1 g, 32 mmol) was added and the milky mixture was stirred at rt overnight. The reaction was diluted with 0.5 M aqueous NaOH (70 mL, 35 mmol), stirred for 10 minutes and extracted with DCM (3 × 20 mL). The organic layers were combined and washed with sat. aq. NaHCO_3_ (3 × 20 mL) and brine (20 mL)., dried over Na_2_SO_4_ and solvents were removed *in vacuo.* Colorless liquid (2.0 g, 100%). ^1^H NMR (CDCl_3_): δ 2.91 (m, 1H), 2.75 (m, 1H), 2.48 (m, 1H), 1.46 (m, 4H), 1.32 (m, 8H), 0.88 (m, 3H). (M+H)^+^ = 143 m/z

**1-aminononan-2-ol (36)**

To a solution of 25% aqueous NH_4_OH (50 mL, 360 mmol) was added dropwise compound **35** (2.0 g, 13 mmol). The reaction was stirred overnight at rt. Solvents were removed *in vacuo*. Yellow oil (2.1 g, 100%). ^1^H NMR (CDCl_3_): δ 3.50 (m, 1H), 2.83 (m, 1H), 2.51 (m, 1H), 1.40 (m, 12H), 0.88 (m, 3H). (M+H)^+^ = 160 m/z

**(4*R*)-*N*-(3-((2-hydroxynonyl)amino)-3-oxopropyl)-2,2,5,5-tetramethyl-1,3-dioxane-4-carboxamide (37)**

To a cooled solution of compound **2** (1g, 3,86 mmol) in DMF (15 mL) were added HOBt (0.67 g, 4.2 mmol), EDC) (0.81 g, 4.2 mmol), DIPEA (0.74 mL, 4.2 mmol) and a solution of amino alcohol **36** (610 g, 3.9 mmol) in DMF (7 mL). The reaction was stirred overnight at rt. The mixture was diluted with EtOAc (100 mL) and 10% citric acid (75 mL). After layer separation, the aqueous layer was extracted with EtOAc (50 mL). The combined organic layers were washed with 10% citric acid (2 × 75 mL), sat. aq. NaHCO_3_ (2 ×75 mL) and brine (2 × 75 mL), dried over Na_2_SO_4_ and solvents were removed *in vacuo.* Yellow oil (1.0 g, 66%). (M+H)^+^ = 401 m/z

**(*R*)-2,2,5,5-tetramethyl-*N*-(3-oxo-3-((2-oxononyl)amino)propyl)-1,3-dioxane-4-carboxamide (38)**

Compound **37** (1.4 g, 3.4 mmol) was dissolved in DCM (35 mL), Dess Martin Periodinane (2.2 g, 5.2 mmol) was added. The yellow solution was stirred at rt for 1 h. The mixture was diluted with Et_2_O (85 mL), washed with 30% Na_2_S_2_O_3_ in sat. aq. NaHCO_3_ (2 × 25 mL), brine (2 × 30 mL), dried over Na_2_SO_4_ and solvents were removed *in vacuo.* The crude was purified by flash chromatography (Heptane / EtOAc / MeOH, from 10 : 90 : 0 to 0 : 99 : 1). Oil (0.9 g, 64%). ^1^H NMR (CDCl_3_): δ 7.05 (s, 1H), 6.37 (bs, 1H), 4.13 (dd, 2H, *J* = 5.2, 18.4), 4.08 (s, 1H), 3.68 (d, 1H, *J* = 12.0), 3.57 (m, 2H), 3.27 (d, 1H, *J* = 11.6), 2.50 (t, 2H, *J* = 6.0), 2.44 (t, 2H, *J* = 7.25), 1.61 (m, 2H), 1.46 (s, 3H), 1.42 (s, 3H), 1.28 (m, 8H), 1.04 (s, 3H), 0.96 (s, 3H), 0.88 (m, 3H). (M+H)^+^ = 399 m/z

**(*R*)-*N*-(2-(5-heptyloxazol-2-yl)ethyl)-2,2,5,5-tetramethyl-1,3-dioxane-4-carboxamide (39)**

Under a N_2_ conditions, to a solution of **38** (200 mg, 0.5 mmol) in THF (4 mL) was added Burgess reagent (441 mg, 1.8 mmol) in a MW vessel and the reaction was heated at the MW 0.35 h, 75 °C at 150W. The mixture was diluted with sat. aq. NaHCO_3_ (20 mL) and EtOAc (20 mL). After layer separation, the waterlayer was extracted with EtOAc (10 mL). The combined organic layers were dried over Na_2_SO_4_ and the solvents were removed *in vacuo.* The crude was purified by flash chromatography (Heptane / EtOAc, from 50 : 50 to 80 : 20). Yellowish oil (72 mg, 36%). ^1^H NMR (CDCl_3_): δ 7.13 (bs, 1H), 6.62 (s, 1H), 4.08 (s, 1H), 3.68 (m, 3H), 3.27 (d, 1H, *J* = 11.6), 2.93 (t, 2H, *J* = 6.4), 2.40 (t, 2H, *J* = 7.2), 1.61 (m, 2H), 1.46 (s, 3H), 1.42 (s, 3H), 1.30 (m, 8H), 1.03 (s, 3H), 0.88 (s, 3H), 0.84 (m, 3H). (M+H)^+^ = 381 m/z

**(*R*)-*N*-(2-(5-heptyloxazol-2-yl)ethyl)-2,4-dihydroxy-3,3-dimethylbutanamide (40; CXP14.19-104)**

Compound prepared following general procedure **B** using compound **38**. Colorless oil (59 mg, 87%).^1^H NMR (CDCl_3_): δ 7.31 (bs, 1H), 6.60 (s, 1H), 3.97 (s, 1H), 3.65 (m, 2H), 3.50 (dd, 2H, *J* = 11.2, 30.8), 2.94 (m, 2H), 2.60 (t, 2H, *J* =7.2), 1.61 (m, 2H), 1.30 (m, 8H), 1.04 (s, 3H), 0.97 (s, 3H), 0.89 (t, 3H, *J* =7.2). (M+H)^+^ = 341 m/z

**(*R*)-*N*-(2-(5-heptylthiazol-2-yl)ethyl)-2,2,5,5-tetramethyl-1,3-dioxane-4-carboxamide (41)**

In a MW vessel was added compound **31** (200 mg, 0.5 mmol) and dissolved in THF (4mL). Lawesson's reagent (610 mg, 1.5 mmol) and K_2_CO_3_ (90 mg, 0.6 mmol) were added and the suspension was heated at the MW 0.25 h, 55 °C 75W. The mixture was diluted with sat. aq. NaHCO_3_ (15 mL) and EtOAc (15 mL). After layer separation the aqueous layer was extracted with EtOAc (2 × 10 mL). The combined organic layers were dried over Na_2_SO_4_. Solvents were removed *in vacuo* and the crude was purified by flash chromatography (Heptane / EtOAc, from 50 : 50 to 30 : 70). Colorless oil (23 mg, 12%). ^1^H NMR (CDCl_3_): δ 7.33 (s, 1H), 7.16 (bs, 1H), 4.08 (s, 1H), 3.68 (m, 3H), 3.27 (d, 1H, *J* = 12.0), 3.15 (t, 2H, *J* = 6.4), 2.77 (t, 2H, *J* = 7.2), 1.63 (m, 2H), 1.45 (s, 3H), 1.42 (s, 3H), 1.30 (m, H), 1.04 (s, 3H), 0.93 (s, 3H), 0.88 (t, 3H, J = 7.2). (M+H)^+^ = 397 m/z

**(*R*)-*N*-(2-(5-heptylthiazol-2-yl)ethyl)-2,4-dihydroxy-3,3-dimethylbutanamide (42; CXP14.19-103)**

Compound prepared following general procedure **B** using compound **41**. Colorless oil (38 mg, 89%).^1^H NMR (CDCl_3_): δ 7.31 (s, 1H), 7.30 (bs, 1H), 4.15 (bs, 1H), 3.97 (s, 2H), 3.65 (m, 2H), 3.50 (m, 2H), 3.13 (t, 2H, *J* = 6.0), 2.77 (t, 2H, *J* = 8.0), 1.63 (qn, 2H, *J* = 7.8), 1.30 (m, 6H), 1.04 (s, 3H), 0.97 (s, 3H), 0.89 (t, 3H, *J* = 7.2). (M+H)^+^ = 357 m/z

**(*R*)-*N*-(2-(5-heptyl-1H-imidazol-2-yl)ethyl)-2,2,5,5-tetramethyl-1,3-dioxane-4-carboxamide (43)**

Compound **38** (200 mg, 0.5 mmol) was dissolved in 1,4-dioxane (2.5 mL) in a sealable tube. After addition of ammonium acetate (970 mg, 12 mmol) the tube was tightly sealed and the reaction was stirred at 105°C in an oil bath for 48 h. After cooling to rt the mixture was diluted with EtOAc (6 mL), filtered and solvents were removed *in vacuo* The crude was purified by flash chromatography (EtOAc / MeOH, from 100 : 0 to 80 : 20). Oil (36 mg, 19%). ^1^H NMR (CDCl_3_): δ 7.14 (bs, 1H), 6.64 (s, 1H), 4.08 (s, 1H), 3.68 (m, 3H), 3.27 (d, 1H, *J* = 11.6), 3.01 (t, 2H, *J* = 6.0), 2.54 (t, 2H, *J* = 7.2), 1.61 (m, 2H), 1.46 (s, 3H), 1.42 (s, 3H), 1.30 (m, 8H), 1.02 (s, 3H), 0.88 (m, 3H), 0.84 (s, 3H). (M+H)^+^ = 380 m/z

**(*R*)-*N*-(2-(5-heptyl-1H-imidazol-2-yl)ethyl)-2,4-dihydroxy-3,3-dimethylbutanamide (44; CXP14.19-112)**

Compound prepared following general procedure **B** using compound **43**. Colorless oil (27 mg, 85%). ^1^H NMR (CDCl_3_): δ 7.31 (s, 1H), 7.30 (bs, 1H), 4.15 (bs, 1H), 3.97 (s, 2H), 3.65 (m, 2H), 3.50 (m, 2H), 3.14 (t, 2H, *J* = 5.6), 2.77 (t, 2H, *J* = 6.8), 1.63 (m, 2H), 1.30 (m, 6H), 1.04 (s, 3H), 0.97 (s, 3H), 0.89 (t, 3H, *J* = 7.2). (M+H)^+^ = 343 m/z

**(R)-2,4-dihydroxy-3,3-dimethyl-N-(prop-2-yn-1-yl)butanamide (45)**

To a solution of D-pantolactone (500 mg, 3.84 mmol) and propargylamine**^.^**HCl (234 mg, 2.56 mmol) in ethanol (2.6 mL), DIPEA (1.12 mL, 6.40 mmol) was added and the mixture was stirred at 75 °C and left overnight. The mixture was allowed to cool down to room temperature, diluted with sat. aq. NH_4_Cl (17.5 mL), H_2_O (2.5 mL) and EtOAc (20 mL). The aqueous phase was extracted with EtOAc (2 x 15 mL). The combined organic phases were dried over Na_2_SO_4_ and concentrated *in vacuo* affording the crude product as an orange oil. Purification by flash column chromatography (heptane / EtOAc = 1: 6), Rf = 0,35 (eluent 1:6) afforded **1** (241 mg, 49%) as a pale orange oil. ^1^H NMR (CDCl_3_): δ = 7.00 (br s, 1H), 4.09-4.08 (dd, 2H, *J* = 4.8 Hz), 3.56-3.53 (app. q, 2H *J* = 12 Hz), 2.94 (br s, 1H), 2.24 (t, 1H, *J=* 4 Hz), 1.69 (br s, 1H) 1.05 (s, 3H), 0,94 (s, 3H).

**(R)-N-(but-3-yn-1-yl)-2,4-dihydroxy-3,3-dimethylbutanamide (46)**

To a solution of D-pantolactone (565 mg, 4.34 mmol) and 3-butynylamine (0.24 mL, 2.89 mmol) in ethanol ( 2.8 mL), DIPEA (0.76 mL, 4.34 mmol) was added and the mixture, a clear solution, was stirred at 60° C. Because conversion was not complete, more D-pantolactone was added; 100 mg after 2 h, 50 mg after 4 h 15 min and 50 mg after 5,5 h. The mixture was allowed to cool down to room temperature, diluted with sat. aq. NH_4_Cl (17.5 mL), H_2_O (2.5 mL) and with EtOAc (20 mL). The aqueous phase was extracted with EtOAc (2 x 15 mL). The combined organic phases were dried over Na_2_SO_4_ and concentrated *in vacuo,* the crude product was afforded as a colorless oil. Purification by flash column chromatography (heptane / EtOAc = 1:1 🡪 1:3 🡪 1:6) afforded **2** (440 mg, 68%) as a colorless oil. ^1^H NMR (CDCl_3_): δ = 7.07 (br s, 1H), 4.06 (d, 1H *J* = 4.4 Hz), 3.67 (d, 1H, *J* = 4.8 Hz), 3.58-3.39 (m, 4H), 3.07 (br s, 1H), 2.44 (dt, 2H, *J* = 4Hz, 2.4 Hz), 2.02(t, 1H, *J* = 2.8), 1.05 (s, 1H), 0.94 (s, 1H).

**1-azidoheptane (47)**

To a suspension of sodium azide (248 mg, 3.82 mmol) in DMSO (8.5 mL), 1-bromoheptane (200 µL, 1.27 mmol) was added. The mixture turned rapidly (15 min) into a clear solution and it was stirred overnight at room temperature. The reaction mixture, a clear solution, was diluted with DCM (20 mL) and H_2_O (10 mL). The organic layer was washed with H_2_O (2 x 10 mL), washed with brine (2 x 10 mL) and was dried over Na_2_SO_4_ affording **4** which was kept in solution (because of explosion hazard). 8 drops were evaporated to measure NMR. ^1^H NMR(CDCl_3_): δ = 3.26 (t, 2H, *J* = 6.8), 1.53-1.65 (m, 2H) 1.21-1.39 (m, 8H), 1.29 (t, 3H, *J* = 6,8 Hz).

**A: (*R*)-*N*-((1-heptyl-1H-1,2,3-triazol-4-yl)methyl)-2,4-dihydroxy-3,3-dimethylbutanamide** and **B: (*R*)-*N*-((1-heptyl-1H-1,2,3-triazol-5-yl)methyl)-2,4-dihydroxy-3,3-dimethylbutanamide** **(48; CXP14.20-017)**

A mixture of **45** (40 mg, 0.20 mmol) and **47** (42 mg, 0.30 mmol) was left in 1 mL DCM at 50 °C, 15 kbar for 4 nights (89 h). Purified by flash column chromatography (EtOAc/ MeOH = 100:1 🡪 100:3), Rf = 0.37 (eluent 100:1) affording **48** (66 mg 103%) as a colorless oil, a mixture of the two regio-isomers with ratio 1:0.74 (A:B). ^1^H NMR(CDCl_3_): δ = 7.56 (s, 1H_B_), 7.51 (s, 1H_A_), 7.41 (br t, 1H_A/B_, *J* = 5.6 Hz), 7.21 (br t, 1H_A/B_, *J* = 5.6 Hz), 4.63- 4.50 (m, 4H_A+B_), 4.33 (t, 2H_A_, *J* = 7.2 Hz), 4.32 (t, 2H_B_, *J* = 7.2 Hz), 4.14-4.03 (m, 3H_A+B_), 3.78 (br d, 1H_A/B_ *J* = 5.6 Hz), 3.61-3.48 (m, 4H_A+B_), 3.31 (br t, 1H_A/B_, *J* = 5.6 Hz), 2.84 (br t, 1H_A/B_, *J* = 5.2 Hz), 1.81-1.93 (m, 4H_A+B,_), 1.39-1.19 (m, 16H_A+B_), 1.03 (s, 3H_B_), 1.02 (s, 3H_A_), 0.96 (s, 3H_B_), 0.93 (s, 3H_A_), 0.87 (app t, 6H_A+B_, *J =* 6.4 Hz)

**A: (*R*)-*N*-(2-(1-heptyl-1H-1,2,3-triazol-4-yl)ethyl)-2,4-dihydroxy-3,3-dimethylbutanamide** and **B: (*R*)-*N*-(2-(1-heptyl-1H-1,2,3-triazol-5-yl)ethyl)-2,4-dihydroxy-3,3-dimethylbutanamide (49; CXP14.20-020)**

A mixture of **46** (40 mg, 0.18 mmol) and **47** (38 mg, 0.27 mmol) was left in 1 mL DCM at 50 °C, 15 kbar for 2 nights (45 h)*.* Purified by flash column chromatography (EtOAc/MeOH = 100:1 → 100:3), Rf = 0.22 (eluent 100:1) affording **49** (13 mg, 22%) as a colorless oil, a mixture of the two regio-isomers with ratio 1:1. ^1^H NMR(CDCl_3_): δ = 7.49 (s, 1H_A/B_), 7.38 (s, 1H_A/B_), 7.28 (br t, 1H_A/B_, *J* = 5.6 Hz), 7.20 (br t, 1H_A/B_, *J* = 6.0 Hz), 4.47 (br d, 1H_A/B_, *J* = 4.8 Hz), 4.34-4.18 (m, 5H_A+B_), 4.18 (br s, 1H_A/B_), 4.05 (d, 1H_A/B_, *J =* 4.4 Hz), 3.97 (d, H_A/B_, *J =* 6.0 Hz), 3.75-3.40 (m, 9H_A+B_), 2.97-2.88 (m, 4H_A+B_), 1.93-1.87 (m, 4H_A+B_), 1.93-1.87 (m, 16H_A+B_), 1.02 (s, 3H_A/B_), 0.99 (s, 3H_A/B_), 0.95 (s, 3H_A/B_), 0.92 (s, 3H_A/B_), 0.88 (t, 3H_A/B_, *J =* 6.8 Hz), 0.87 (t, 3H_A/B_, *J =* 6.8 Hz).

**(*R*)-*N*-(2-aminoethyl)-2,4-dihydroxy-3,3-dimethylbutanamide (50)**

A solution of D-pantolactone (2.50 g, 19.21 mmol) in ethane-1,2-diamine (5.83 mL, 96.00 mmol) was stirred at 55 °C overnight. The mixture was concentrated *in vacuo* (cont. 50 °C, 45 min). Purification was performed by flash column chromatography (chloroform /methanol/NH_4_OH = 325:185:0 🡪 325:200:0 🡪 325:200:50), Rf = 0.51 (eluent 1:6). affording **9** (2.56 g, 66%) as a pale orange oil. ^1^H NMR(MeOD): δ = 3.91 (s, 1H), 3.44 (dd, 2H *J =* 6.8 Hz, 18 Hz), 3.33-3.23 (m, 2H), 2.76-2.71 (m, 2H), 0.94 (d, 6H, *J* = 1.6 Hz).

**(*R*)-*N*-(2-azidoethyl)-2,4-dihydroxy-3,3-dimethylbutanamide (51)**

To a mixture of **50** (1.00 g, 4.98 mmol), NaHCO_3_ (1.26 g, 14.94 mmol) and CuSO_4_·5H_2_O (9.24 mg, 0.05 mmol) in a mixture of THF (12.5 mL) and water (12.5 mL), 1H-imidazole-1-sulfonyl azide hydrochloride (1.28 g, 5.97 mmol)was added, and the mixture was stirred at room temperature overnight. The mixture was concentrated *in vacuo*, diluted with H_2_O (12.5 mL), made acidic with HCl and extracted with EtOAc (3 x 25 mL). The combined organic layers were dried over Na_2_SO_4_, concentrated *in vacuo* affording **10** (1.08 g, 85%) as a colorless oil. ^1^H NMR (CDCl_3_) δ = 7.08 (s, 1H), 4.07 (d, 1H, *J* = 4.0 Hz), 3.76 (br d, 1H, *J* = 4.8 Hz), 3.60-3.40 (m, 6H), 3.03 (br s, 1H), 1.05 (s, 3H), 0.94 (s, 3H).

**A:** **(R)-N-((1-heptyl-1H-1,2,3-triazol-4-yl)methyl)-2,4-dihydroxy-3,3-dimethylbutanamide** and **B: (R)-N-((1-heptyl-1H-1,2,3-triazol-4-yl)methyl)-2,4-dihydroxy-3,3-dimethylbutanamide** **(52; CXP14.20-029)**

A mixture of **51** (80 mg, 0.31 mmol) and 1-nonyne (0.08 mL, 0.47 mmol) was left in DCM (1.5 mL) at 50 °C, 15 kbar for 3 nights (~73 h)*.* Purified by flash column chromatography (EtOAc/MeOH = 100:3 🡪 100:5), Rf = 0.20 (eluent 100:1) affording **12b** (38 mg, 35%) as a colorless oil, a mixture of the two regio-isomers with ratio 1:1.35 (A: B). ^1^H NMR(CDCl_3_): δ = 7.46 (s, 1H_B_), 7.33 (s, 1H_A_), 7.30 (br t, 1H_A/B_, *J* = 6.4 Hz), 7.22 (br t, 1H_A/B_, *J* = 6.4 Hz), 4.48 (t, 2H_A_, *J* = 6.4 Hz), 4.40-4.35 (m, 2H_B_), 4.18-4.09 (m, 2H_A+B_), 4.01 (t, 2H_A/B_, *J* = 6.4 Hz), 3.88-3.72 (m, 4H _A+B_), 3.56-3.47 (m, 4H _A+B_), 3.35 (br s, 1H_A/B_), 3.30 (br s, 1H_A/B_), 2.69 (t, 3H_A_, *J* = 8.0 Hz), 2.62 (t, 3H_B_, *J* = 7.6 Hz), 1.72-1.57 (m, 4H_A+B_), 1.43-1.21 (m, 16H_A+B_), 0.99 (app s, 6H_A+B_), 0.94-0.84 (m, 12H_A+B_, *J* = 6.8 Hz).

**1-(piperazin-1-yl)octan-1-one (53)**

**Octanoic acid** (663 mg, 4.60 mmol) and HOBt (640 mg, 4.60 mmol) were suspended in chloroform (9.6 mL). DCC (960 mg, 4.60 mmol) was added followed by peptide grade DMF (1.1 mL). The mixture, a very thick white suspension, was stirred for 30 min at room temperature. Then the mixture was transferred slowly to a cooled solution of piperazine (2.0 g, 22.99 mmol) in chloroform (23 mL) and it was stirred at room temperature. After 2 h the white suspension was filtered off and the filtrate was extracted with 1 M aqueous HCl solution (3 x 24 mL). The combined aqueous layers were basified till pH 8.1 with 1 M aq NaOH solution. The white suspension was extracted with EtOAc (4 x 25 mL), washed with brine (4 x 20 mL), dried over Na2SO4 and concentrated *in vacuo* affording **53** (487 mg, 49%) as a white solid. ^1^H NMR (CDCl_3_): δ = 3.69-3.55 (m, 2H), 3.48-3.37 (m, 2H), 2.80-2.89 (m, 4H) 2.13-2.35 (m, 2H), 1.53-1.79 (m, 2H), 1.21-1.40 (m, 8H), t = 0.88 (t, 3H *J =* 6.8 Hz).

**1-(4-((4*R*)-5,5-dimethyl-2-phenyl-1,3-dioxane-4-carbonyl)piperazin-1-yl)octan-1-one (54)**

To a solution of (4*R*)-5,5-dimethyl-2-phenyl-1,3-dioxane-4-carboxylic acid (119 mg, 0.47 mmol) in DCM (1.2 mL), were added HOBt (80 mg, 0.51 mmol), DIPEA (0.089 mL, 0.51 mmol), EDC (98 mg, 0.51 mmol) and **53** (100 mg, 0.47 mmol) in DCM (1.2 mL). The mixture was reacted according to the general procedure affording the crude product **54** (178mg, 80%) as a white solid.

**(*R*)-1-(4-(2,4-dihydroxy-3,3-dimethylbutanoyl)piperazin-1-yl)octan-1-one (55; CXP14.20-027)**

To a solution of **54** (177 mg, 0.37 mmol) in 2-propanol (8.0 mL) was added palladium on carbon 10 w% (60 mg, 0.06 mmol). Hydrogen gas was bubbled through the black suspension and the mixture was stirred at 40 °C. Because of low conversion after 1 h and 45 min there was added again palladium on carbon 10 w/w% (60 mg). After 40 min conversion was completed. Then the mixture was filtered over Celite and rinsed with IPA (2 x 10 mL, 1 x 5mL) and the residu was concentrated *in vacuo*. Purification by flash column chromatography (EtOAc/MeOH = 100:2 🡪 100:5 🡪 100:10) Rf = 0.15 (eluent 100: 2) affording **55** (75 mg, 56%) as a white solid. ^1^H NMR(CDCl_3_): δ = 4.47 (d, 1H *J =* 9.2 Hz), 3.94-3.68 (m, 2H), 3.68-3.35 (m, 9H), 2.42-2.25 (m, 3H), 1.72-1.57 (m, 2H), 1.39-1.21 (m, 8H), 0.96 (s, 3H), 0.94 (s, 3H), 0.88 (t, 3H, *J* = 6.8 Hz).

**(*R*)-2,4-dihydroxy-3,3-dimethylbutanamide (56)**

Pantolactone (3.02 g, 23.23 mmol) was added to liquid ammonia (24 mL, 960 mmol) at -60 °C. After 1 hour the pantolactone was completely dissolved and the solution was kept at -60 °C for another hour before the mixture was allowed to warm up to room temperature overnight. The excess of ammonia was evaporated to gain **56** as a white solid (3.22 g, 94%). R_f_ = 0.30 (DCM/MeOH = 9:1); ^1^H-NMR (CD_3_OD): δ 3.90 (s, 1H); 3.49 (d, 1H, *J =* 10.9 Hz); 3.39 (d, 1H, *J =* 10.9 Hz).

**(*R*)-2,2,5,5-tetramethyl-1,3-dioxane-4-carboxamide (57)**

To an ice-cooled solution of **56** (3.22 g, 21.9 mmol) in acetone (100 mL) was added subsequently 2-methoxyprop-1-ene (6.30 mL, 65.8 mmol) and PTSA (0.17 g, 0.88 mmol). The temperature was raised to room temperature after 15 minutes by removing the ice bath. After 45 minutes, a solution of sat. aq. NaHCO_3_ (1.6 mL) was added to the mixture and after evaporation of the solvent the product was purified by flash column chromatography (EtOAc/heptane = 2:1 -> 1:1). **57** was obtained as a white solid (3.527 g, 86%). R_f_ = 0.71 (DCM/MeOH = 9:1); ^1^H-NMR (CDCl_3_): δ 6.47 (s, 1H); 5.70 (s, 1H); 4.10 (s, 1H); 3.70 (d, 1H, *J =* 11.7 Hz); 3.31 (d, 1H, *J =* 11.7 Hz); 1.48 (s, 3H); 1.44 (s, 3H); 1.08 (s, 3H); 1.06 (s, 3H).

**1H-benzo[d][1,2,3]triazole-1-carbaldehyde (58)**

1H-benzo[d][1,2,3]triazole (3.32 g, 27.9 mmol) was dissolved in dry DCM (60 mL) at 0 °C. Formic acid (1.26 mL, 33.4 mmol) was added followed by DCC (8.05 g, 39.0 mmol). The temperature was slowly raised to room temperature after addition. After 21 hours the reaction mixture was filtered and the solvent was evaporated. The remaining white solid was triturated in 30% EtOAc in Et_2_O. The mixture was filtered and the solvent was evaporated. The product was dissolved in Et_2_O (15 mL) at 40 °C, and upon cooling to room temperature the product precipitated from the solution. Subsequently the suspension was cooled to 0 °C to ensure complete precipitation of the product. The product was filtered off as a white solid (1.85 g, 45%). ^1^H-NMR (CDCl_3_): δ 9.87 (s, 1H); 8.26 (m, 1H); 8.17 (m, 1H); 7.72 (m, 1H); 7.58 (m, 1H).

**(*R*)-*N*-formyl-2,2,5,5-tetramethyl-1,3-dioxane-4-carboxamide (59)**

To an ice-cooled solution of **57** (1.20 g, 6.40 mmol) in THF (20 mL) was added a 1.6M solution of n-BuLi in hexane (4.40 mL, 7.04 mmol). The mixture was stirred for 15 min at 0 °C before **58** (1.13 g, 7.68 mmol) was added. The mixture was allowed to warm up to room temperature in 2 hours. The mixture was diluted with Et_2_O (60 mL) and quenched with sat. aq. NaHCO_3_ (15 mL). The organic phase was washed with sat. aq. NaHCO_3_ (20 mL) and brine (20 mL), dried and filtrated before evaporation of the solvent. The product was purified by flash column chromatography (EtOAc/heptane = 1:7 -> 1:5 -> 1:3). **59** was obtained as a white solid (989 mg, 72%). R_f_ = 0.32 (EtOAc/Heptane = 4:1); ^1^H-NMR (CDCl_3_): δ 9.15 (d, 1H, *J =* 10.41); 8.90 (s, 1H); 4.20 (s, 1H); 3.7 (d, 1H, *J =* 11.8 Hz); 3.33 (d, 1H, *J =* 11.8 Hz); 1.49 (s, 3H); 1.45 (s, 3H); 1.06 (s, 3H); 1.05 (s, 3H).

**(R*,E*)-tert-butyl 3-(2,2,5,5-tetramethyl-1,3-dioxane-4-carboxamido)acrylate (60)** and **(R,*Z*)-tert-butyl 3-(2,2,5,5-tetramethyl-1,3-dioxane-4-carboxamido)acrylate (61)**

A solution of **59** (250 mg, 1.16 mmol) in toluene (10 mL) was treated with tert-butyl 2-(triphenylphosphoranylidene)acetate (1.31 g, 3.48 mmol) at 95 °C. The mixture was stirred of 16 hours before evaporation of the solvent. The product was purified by flash column chromatography (EtOAc/heptane = 1:6 -> 1:4 -> 1:2). Two products were isolated (342 mg). *E-*isomer **60** was obtained as a white solid (235 mg, 64%) : R_f_ = 0.26 (EtOAc/Heptane = 1:4); 1H-NMR (CDCl_3_): δ 8.31 (d, 1H, *J =* 11.5 Hz); 7.86 (dd, 1H, *J =* 11.8 Hz, *J =* 14.1 Hz); 5.53 (d, 1H, *J =* 14.2 Hz); 4.19 (s, 1H); 3.71 (d, 1H, *J =* 11.7 Hz); 3.31 (d, 1H, *J =* 11.7 Hz); 1.51 (s, 3H); 1.48 (s, 9H); 1.45 (s, 3H); 1.05 (s, 3H); 1.00 (s, 3H).

*Z*-isomer **61** was obtained as a white solid (107 mg, 30%): R_f_ = 0.45 (EtOAc/Heptane = 1:4); ^1^H-NMR (CDCl_3_): δ 11.2 (d, 1H, *J =* 11.3 Hz); 7.35 (dd, 1H, *J =* 8.95 Hz, *J =* 11.5 Hz); 5.08 (d, 1H, *J =* 9.05); 4.20 (s, 1H); 3.73 (d, 1H, *J =* 11.7 Hz); 3.33 (d, 1H, 11.7 Hz); 1.58 (s, 3H); 1.49 (s, 9H); 1.47 (s, 3H); 1.05 (s, 6H).

**(*R*,*E*)-tert-butyl 3-(2,4-dihydroxy-3,3-dimethylbutanamido)acrylate(62)**

A solution of **60** (400 mg, 1.27 mmol) in a mixture of methanol/water (ratio 2:1, 12 mL) was treated with PTSA (19.4 mg, 0.10 mmol). After 18 hours, the reaction was still not completed, so for the second time PTSA (15.0 mg, 0.09 mmol) was added. The mixture was stirred of another 4 hours before it was diluted with water (80 mL) and EtOAc (80 mL). The organic phase was separated and washed with sat. aq. NaHCO_3_ (80 mL), brine (80 mL) and dried over Na_2_SO_4_. After evaporation of the solvent the product was obtained as a white solid (325 mg, 93%). R_f_ = 0.32 (EtOAc/Heptane = 3:1); ^1^H-NMR (CDCl_3_): δ 8.78 (d, 1H, *J =* 11.9 Hz); 7.89 (dd, 1H, *J =* 11.9 Hz, *J =* 14.2 Hz); 5.50 (d, 1H, *J* = 14.2 Hz); 4.18 (s, 1H); 3.61 (d, 1H, *J =* 10.9 Hz); 3.58 (d, 1H, *J =* 10.9 Hz); 2.50 (s, 1H); 1.49 (s, 9H); 1.05 (s, 3H); 0.98 (s, 3H).

**(*R*,*E*)-3-(2,4-dihydroxy-3,3-dimethylbutanamido)acrylic acid (63)**

**62** (225 mg, 0.82 mmol) was dissolved in formic acid (3 mL) and stirred for 2 hours. The solvent was evaporated and once co-evaporated with toluene. The remainder was dissolved in MeOH (5 mL) and K_2_CO_3_ (124 mg, 0.90 mmol) was added. After 1 hour, the mixture was diluted with water (30 mL) and EtOAc (40 mL). The mixture was acidified to pH 3 by addition of 0.1M aq. HCl (10 mL). The aqueous layer was extracted with EtOAc (2 × 50 mL). The organic phases were combined and washed with brine (50 mL), dried over Na_2_SO_4_ and filtered. After the solvent was evaporated the product was purified by flash column chromatography (EtOAc/heptane = 2:1 -> 4:1 -> 8:1, all with 0.5% AcOH). The product was obtained as a colourless oil (88 mg, 50%). R_f_ = 0.17 (EtOAc/Heptane = 3:1); ^1^H-NMR (CDCl_3_): δ 7.93 (d, 1H, *J =* 14.2 Hz); 5.69 (d, 1H, *J =* 14.2 Hz); 4.01 (s, 1H); 3.49 (d, 1H, *J =* 10.8 Hz); 3.38 (d, 1H, *J =* 10.8 Hz); 0.94 (s, 3H); 0.93 (s, 3H).

**(*R*,*E*)-*N*-(3-(heptylamino)-3-oxoprop-1-en-1-yl)-2,4-dihydroxy-3,3-dimethylbutanamide (64; CXP14.1-057)**

A ice-cooled solution of **63** (44 mg, 0.20 mmol) in DMF (2 mL) was treated subsequently with HOBt.H_2_O (37.2 mg, 0.24 mmol), EDC.HCl (46.6 mg, 0.24 mmol), 1-aminoheptane (39 μL, 0.26 mmol) and DIPEA (42 μL, 0.24 mmol). When the starting material was completely converted, the mixture was diluted with sat. aq. NH_4_Cl (8 mL) and water (2 mL). The mixture was extracted with EtOAc (2 × 15 mL). The organic phases were combined and washed with sat. aq. NH_4_Cl (5 mL), sat. aq. NaHCO_3_ (5 mL) and brine (5 mL). The organic phase was dried over Na_2_SO_4_, filtered and the solvent evaporated. Purification was performed by flash column chromatography (MeOH/DCM = 3% -> 6% -> 10%). The product was obtained as a white solid (32 mg, 50%). R_f_ = 0.35 (MeOH/DCM = 5%); ^1^H-NMR (CDCl_3_): δ 7.80 (d, 1H, *J =* 14.0 Hz); 5.89 (d, 1H, *J =* 14.0 Hz); 4.00 (s, 1H); 3.48 (d, 1H, *J =* 10.9 Hz); 3.38 (d, 1H, *J =* 10.9 Hz); 3.21 (t, 2H, *J =* 7.1 Hz); 1.51 (t, 2H, *J =* 7.1 Hz); 1.32 (m, 9H); 0.93 (s, 6H).

**(*R*,*Z*)-tert-butyl 3-(2,4-dihydroxy-3,3-dimethylbutanamido)acrylate (65)**

To a solution of **61** (279 mg, 0.89 mmol) in a mixture of MeOH/water (ratio 3:1**,** 10 mL) was added PTSA (25 mg, 0.13 mmol). The addition of PTSA (25 mg, 0.13 mmol) was repeated two times (after 12 hours and after 20 hours) to push the reaction to full conversion (after a total reaction time of 36 hours. The mixture was diluted with water (80 mL) and EtOAc (120 mL) and the layers were separated. The organic phase was washed with sat. aq. NaHCO_3_ (80 mL), brine (80 mL) and dried over Na_2_SO_4_. The product was gained as a colourless oil (235 mg, 97%) after evaporation of the solvent. R_f_ = 0.40 (EtOAc/Heptane = 1:1); ^1^H-NMR (CDCl_3_): δ 7.34 (d, 1H, *J =* 9.1 Hz); 5.07 (d, 1H, *J =* 9.1 Hz); 4.04 (s, 1H); 3.49 (d, 1H, 10.8 Hz); 3.40 (d, 1H, *J =* 10.8 Hz); 1.5 (s, 9H); 0.93 (s, 6H).

**(*R*,*Z*)-3-(2,4-dihydroxy-3,3-dimethylbutanamido)acrylic acid (66)**

**65** (50 mg, 0.33 mmol) was dissolved in formic acid (2 mL) and stirred for 2 hours. The solvent was evaporated and once co-evaporated with toluene. The remainder was dissolved in MeOH (5 mL) and K_2_CO_3_ (124 mg, 0.90 mmol) was added. After 1 hour, the mixture was diluted with water (50 mL) and EtOAc (50 mL). The mixture was acidified to pH = 3 by addin 0.1M aqueous HCl (10 mL). The aqueous layer was extracted with EtOAc (2 × 50 mL). The organic phases were combined and washed with brine (70 mL), dried over Na_2_SO_4_ and filtered. After the solvent was evaporated the crude product was obtained as a colourless oil (56 mg, 78%). R_f_ = 0.74 (DCM/MeOH = 9:1); ^1^H-NMR (CD_3_OD): δ 7.43 (d, 1H, *J =* 8.9 Hz); 5.16 (d, 1H, *J =* 8.9 Hz); 4.04 (s, 1H); 3.49 (d, 1H, *J =* 10.8 Hz); 3.40 (d, 1H, *J =* 10.8 Hz); 0.93 (s, 6H).

**(*R*,*Z*)-*N*-(3-(heptylamino)-3-oxoprop-1-en-1-yl)-2,4-dihydroxy-3,3-dimethylbutanamide (67; CXP14.1-080)**

An ice-cooled solution of **66** (56 mg, 0.26 mmol) in DMF (3 mL) was treated with subsequently HOBt.H_2_O (47.2 mg, 0.31 mmol), EDC.HCl (59.3 mg, 0.31 mmol), 1-aminoheptane (50 μL, 0.34 mmol) and DIPEA (54 μL, 0.31 mmol). When the starting material was fully converted the mixture was diluted with sat. aq. NH_4_Cl (12 mL) and water (3 mL). The mixture was extracted with EtOAc (2 × 20 mL). The organic phases were combined and washed with sat. aq. NH_4_Cl (8 mL), sat. aq. NaHCO_3_ (8 mL) and brine (8 mL). The organic phase was dried over Na_2_SO_4_, filtered and the solvent evaporated. Purification was performed by flash column chromatography (MeOH/DCM = 3% -> 6% -> 10%). The product was co-evaporated with Et_2_O (10 mL) and after evaporation of the solvent **67** was obtained as a orange oil (41 mg, 49%). R_f_ = 0.65 (DCM/MeOH = 19:1); ^1^H-NMR (CD_3_OD): δ 7.20 (d, 1H, *J =* 9.0 Hz); 5.18 (d, 1H, *J =* 9.0 Hz); 3.50 (d, 1H, *J =* 10.8 Hz); 3.40 (d, 1H, *J =* 10.8 Hz); 3.18 (t, 2H, *J =* 7.1 Hz); 1.51 (m, 2H); 1.32 (m, 8H); 0.94 (s, 6H).

**(4*R*)-*N*-(2-(heptylcarbamoyl)phenyl)-5,5-dimethyl-2-phenyl-1,3-dioxane-4-carboxamide (68)**

To a cooled (4 °C) solution of (4*R*)-5,5-dimethyl-2-phenyl-1,3-dioxane-4-carboxylic acid (200 mg, 0.78 mmol) in DMF (4 mL) were added DIPEA (0.41 mL, 2.34 mmol), 2-amino-*N*-heptylbenzamide (382 mg, 1.56 mmol) and PyBOP (527 mg, 1.01 mmol). The mixture was allowed to warm to room temperature and stirred for 16 h. Next, the mixture was diluted with Et_2_O (15 mL) and 10% aqueous citric acid (10 mL). The layers were mixed and separated. The aqueous layer was extracted with Et_2_O (10 mL). The combined organic layers were washed with 10% aqueous citric acid (2 × 10 mL), sat. aq. NaHCO_3_ (2 × 10 mL) and brine (10 mL), dried over Na_2_SO_4_ and concentrated *in vacuo.* The crude product was purified by flash column chromatography (DCM:EtOAc = 20:1 with 0.5% (v/v) Et_3_N) affording **68** as a yellow oil (299 mg, 80%). ^1^H NMR (CDCl_3_): δ 11.51 (s, 1H), 8.63 (dd, 1H, *J* = 8.4, 1.0 Hz), 7.82 (dd, 1H, *J* = 8.3, 1.3 Hz), 7.49 – 7.34 (m, 5H), 7.12 – 7.07 (m, 1H), 6.06 (br s, 1H), 4.24 (s, 1H), 3.77 (s, 2H), 3.50 – 3.32 (m, 2H), 1.62 – 1.55 (m, 2H), 1.38 – 1.26 (m, 8H), 1.24 (s, 3H), 1.15 (s, 3H), 0.90 (m, 3H).

**(*R*)-2-(2,4-dihydroxy-3,3-dimethylbutanamido)-*N*-heptylbenzamide (69; CXP14.14-016)**

To a solution of **68** (299 mg, 0.62 mmol) in 2-propanol (31 mL) was added palladium on carbon 10 wt% (66.1 mg, 0.031 mmol). ). Hydrogen gas was bubbled through the black suspension. The mixture was warmed to 35 °C and stirred for 1 h. Then the mixture was filtered over Celite and rinsed with 2-propanol (2 × 10 mL, 1 × 5mL) and the filtrate was concentrated *in vacuo*. The crude product was purified by flash column chromatography (EtOAc:MeOH = 98:2 🡪 90:10) affording **69** (84 mg, 37%) as a glassy solid. 1H NMR(CDCl_3_): δ 11.40 (s, 1H), 8.57 (dd, 1H, *J* = 8.4, 0.9 Hz), 7.64 – 7.32 (m, 2H), 7.12 (td, 1H, *J* = 7.7, 1.1 Hz), 6.19 (br s, 1H), 4.18 (d, 1H, *J* = 5.6 Hz), 3.73 (d, 1H, *J* = 5.6 Hz), 3.68 – 3.50 (m, 2H), 3.50 – 3.33 (m, 2H), 3.01 (t, 1H, *J* = 5.8 Hz), 1.65 – 1.56 (m, 2H), 1.43 – 1.21 (m, 8H), 1.08 (s, 3H), 1.02 (s, 3H), 0.94 – 0.82 (m, 3H).

**methyl 3-((4*R*)-5,5-dimethyl-2-phenyl-1,3-dioxane-4-carboxamido)benzoate (70)**

To a cooled (4 °C) solution of (4*R*)-5,5-dimethyl-2-phenyl-1,3-dioxane-4-carboxylic acid (250 mg, 0.97 mmol) in DMF (5 mL) were added DIPEA (0.54 mL, 3.12 mmol), methyl 3-aminobenzoate (183 mg, 0.97 mmol) and PyBOP (557 mg, 1.07 mmol). The mixture was allowed to warm to room temperature and stirred for 1.5 h. Next, the mixture was diluted with Et_2_O (15 mL) and 10% aqueous citric acid (10 mL). The layers were mixed and separated. The aqueous layer was extracted with Et_2_O (10 mL). The combined organic layers were washed with 10% aqueous citric acid (2 × 10 mL), sat. aq. NaHCO_3_ (2 × 10 mL) and brine (10 mL), dried over Na_2_SO_4_ and concentrated *in vacuo.* The crude product was purified by flash column chromatography (heptane:EtOAc = 5:1 with 0.5% (v/v) Et_3_N) affording **70** as a colourless oil (230 mg, 64%). ^1^H NMR (CDCl_3_): δ 11.51 (s, 1H), 8.63 (dd, 1H, *J* = 8.4, 1.0 Hz), 7.82 (dd, 1H, *J* = 8.3, 1.3 Hz), 7.49 – 7.34 (m, 5H), 7.12 – 7.07 (m, 1H), 6.06 (br s, 1H), 4.24 (s, 1H), 3.77 (s, 2H), 3.50 – 3.32 (m, 2H), 1.62 – 1.55 (m, 2H), 1.38 – 1.26 (m, 8H), 1.24 (s, 3H), 1.15 (s, 3H), 0.90 (m, 3H).

**3-((4*R*)-5,5-dimethyl-2-phenyl-1,3-dioxane-4-carboxamido)benzoic acid (71)**

To a solution of **70** (180 mg, 0.49 mmol) in MeCN (5 mL) was added dropwise a 1M solution of sodium hydroxide (1 mL, 1 mmol). The mixture was warmed to 40 °C and stirred for 16 h. Next, the mixture was diluted with EtOAc (10 mL) and water (10 mL) and acidified to pH = 3 using 1M aq. HCl. The layers were separated and the aqueous layer was extracted with EtOAc (10 mL). The combined organic layers were washed with brine (2 × 10 mL), dried over Na_2_SO_4_ and concentrated *in vacuo* to **71** as a white foam (160 mg, 90%). ^1^H NMR (CDCl_3_): δ 8.30 (s, 1H), 8.07 (t, 1H, *J* = 1.8 Hz), 7.98 (ddd, 1H, *J* = 8.1, 2.2, 1.0 Hz), 7.90 – 7.81 (m, 1H), 7.57 (dq, 2H, *J* = 6.1, 2.4 Hz), 7.51 – 7.39 (m, 4H), 5.61 (s, 1H), 4.29 (s, 1H), 3.78 (q, 2H, *J* = 11.4 Hz), 1.23 (s, 3H), 1.19 (s, 3H).

**(4*R*)-*N*-(3-(heptylcarbamoyl)phenyl)-5,5-dimethyl-2-phenyl-1,3-dioxane-4-carboxamide (72)**

To a cooled (4 °C) solution of **71** (160 mg, 0.44 mmol) in DMF (2 mL) were added DIPEA (0.084 mL, 0.48 mmol), heptylamine (0.071 mL, 0.48 mmol) and PyBOP (250 mg, 0.48 mmol). The mixture was allowed to warm to room temperature and stirred for 1 h. Next, the mixture was diluted with Et_2_O (10 mL) and 10% aqueous citric acid (5 mL). The layers were mixed and separated. The aqueous layer was extracted with Et_2_O (5 mL). The combined organic layers were washed with 10% aqueous citric acid (2 × 5 mL), sat. aq. NaHCO_3_ (2 × 5 mL) and brine (5 mL), dried over Na_2_SO_4_ and concentrated *in vacuo*, affording **72** as a colourless oil (190 mg, 93%). ^1^H NMR (CDCl_3_): δ 8.29 (s, 1H), 7.86 (t, 1H, *J* = 1.8 Hz), 7.76 (ddd, 1H, *J* = 8.1, 2.2, 1.1 Hz), 7.58 – 7.52 (m, 2H), 7.52 – 7.42 (m, 4H), 7.38 (t, 1H, *J* = 7.9 Hz), 6.12 (br s, 1H), 5.60 (s, 1H), 4.26 (s, 1H), 3.77 (q, 2H, *J* = 11.4 Hz), 3.47 – 3.32 (m, 2H), 1.65 – 1.55 (m, 2H), 1.43 – 1.24 (m, 8H), 1.22 (s, 3H), 1.18 (s, 3H), 0.94 – 0.84 (m, 3H).

**(*R*)-3-(2,4-dihydroxy-3,3-dimethylbutanamido)-*N*-heptylbenzamide (73; CXP14.14-024)**

To a solution of **72** (190 mg, 0.42 mmol) in 2-propanol (20 mL) was added palladium on carbon 10 wt% (45 mg, 0.042 mmol). ). Hydrogen gas was bubbled through the black suspension. The mixture was warmed to 35 °C and stirred for 3 h. Then the mixture was filtered over Celite and rinsed with 2-propanol (2 × 10 mL) and the filtrate was concentrated *in vacuo*. The crude product was purified by flash column chromatography (Et_2_O:MeOH = 99:1  98:2) affording **73** (129 mg, 84%) as a colourless oil. ^1^H NMR(CDCl_3_): δ 8.84 (s, 1H), 7.88 (t, 1H, *J* = 1.8 Hz), 7.71 (ddd, 1H, *J* = 8.0, 2.2, 1.0 Hz), 7.54 – 7.41 (m, 1H), 7.36 (t, 1H, *J* = 7.9 Hz), 6.37 (t, 1H, *J* = 5.6 Hz), 4.72 (d, 1H, *J* = 4.4 Hz), 4.17 (d, 1H, *J* = 4.2 Hz), 3.73 – 3.50 (m, 2H), 3.48 – 3.38 (m, 2H), 3.34 (br s, 1H), 1.61 (p, 2H, *J* = 7.5 Hz), 1.46 – 1.22 (m, 8H), 1.07 (s, 3H), 0.99 (s, 3H), 0.94 – 0.78 (m, 3H).

**(*R*)-3-(2,4-dihydroxy-3,3-dimethylbutanamido)-*N*-heptylbenzamide (74)**

To a solution of **16** (500 mg, 1.89 mmol) in DMF (8 mL) were added NH_4_Cl (557 mg, 10.41 mmol) and sodium azide (677, 10.41 mmol). The white suspension was heated to 120 °C and stirred for 16 h. After cooling to room temperature, the mixture was diluted with Et_2_O (30 mL) and water (50 mL). The mixture was acidified to pH = 3 using 1M HCl. The layers were mixed and separated and the aqueous phase was extracted with Et_2_O (2 × 30 mL). The combined organic layers were washed with brine (30 mL), dried over Na_2_SO_4_ and concentrated *in vacuo*. The crude product was purified by flash column chromatography (EtOAc:MeOH = 9:1 🡪 3:1) affording **74** (312 mg, 74%) as a white solid. ^1^H NMR(CDCl_3_): δ 7.25 – 7.19 (m, 1H), 4.16 (s, 1H), 3.93 – 3.74 (m, 2H), 3.74 – 3.66 (m, 1H), 3.39 – 3.17 (m, 3H), 1.47 (s, 3H), 1.43 (s, 3H), 1.05 (s, 3H), 0.86 (s, 3H).

**(*R*)-*N*-(2-(2-heptyl-2H-tetrazol-5-yl)ethyl)-2,2,5,5-tetramethyl-1,3-dioxane-4-carboxamide (75)** and **(*R*)-*N*-(2-(1-heptyl-1*H*-tetrazol-5-yl)ethyl)-2,2,5,5-tetramethyl-1,3-dioxane-4-carboxamide (76)**

To a solution of **74** (93 mg, 0.33 mmol) in THF (3 mL) were added Et_3_N (0.092 mL, 0.66 mmol) and 1-iodoheptane (0.067, 0.410 mmol). The resulting mixture was heated to 60 °C and stirred for 16 h. After cooling to room temperature, the mixture was diluted with EtOAc (10 mL). The layers were mixed and separated and organic phase was sat. aq. NH_4_Cl (2 × 5 mL) and brine (5 mL), dried over Na_2_SO_4_ and concentrated *in vacuo*. The crude product was purified by flash column chromatography (heptane:EtOAc = 2:1 🡪 1:4) affording **75** (80 mg, 64%) as a colourless oil and **76** (30 mg, 24%) as a colourless oil.

**(*R*)-*N*-(2-(2-heptyl-2*H*-tetrazol-5-yl)ethyl)-2,4-dihydroxy-3,3-dimethylbutanamide (77; CXP14.23-017)**

To a solution of **75** (80 mg, 0.21 mmol) in MeCN (1.5 mL) was added 2M aqueous HCl (1.1 mL, 2.2 mmol). The resulting mixture was stirred for 3 h and then diluted with EtOAc (10 mL) and sat. aq. NH_4_Cl (5 mL). The layers were mixed and separated and the aqueous phase was extracted with EtOAc (10 mL). The combined organic layers were washed with sat. aq. NaHCO_3_ (2 × 5 mL) and brine (5 mL), dried over Na_2_SO_4_ and concentrated *in vacuo* and stripped twice with DCM (5 mL), affording **77** (72 mg, 99%) as a waxy white solid. ^1^H NMR(CDCl_3_): 7.20 (br s, 1H), 4.56 (t, 2H, *J* = 7.2 Hz), 4.00 (d, 1H, *J* = 5.5 Hz), 3.80 (ddd, 1H, *J* = 13.6, 6.8, 5.7 Hz), 3.72 – 3.61 (m, 2H), 3.55 – 3.46 (m, 2H), 3.41 (d, 1H, *J* = 5.8 Hz), 3.13 (ddd, 2H, *J* = 6.8, 5.7, 1.2 Hz), 1.99 (p, 2H, *J* = 7.2 Hz), 1.36 – 1.22 (m, 8H), 1.02 (s, 3H), 0.93 (s, 3H), 0.91 – 0.85 (m, 3H).

**(*R*)-*N*-(2-(1-heptyl-1*H*-tetrazol-5-yl)ethyl)-2,4-dihydroxy-3,3-dimethylbutanamide (78; CXP14.23-018)**

To a solution of **76** (30 mg, 0.08 mmol) in MeCN (1 mL) was added 2M aqueous HCl (0.41 mL, 0.82 mmol). The resulting mixture was stirred for 3 h and then diluted with EtOAc (10 mL) and sat. aq. NH_4_Cl (5 mL). The layers were mixed and separated and the aqueous phase was extracted with EtOAc (10 mL). The combined organic layers were washed with sat. aq. NaHCO_3_ (2 × 5 mL) and brine (5 mL), dried over Na_2_SO_4_ and concentrated *in vacuo* and stripped twice with DCM (5 mL), affording **78** (26 mg, 97%) as a white solid. ^1^H NMR(CDCl_3_): 7.48 (br s, 1H), 4.30 – 4.22 (m, 2H), 4.00 (d, 1H, *J* = 5.5 Hz), 3.87 (d, 1H, *J* = 5.4 Hz), 3.84 (m, 1H), 3.52 – 3.44 (m, 2H), 3.25 (t, 1H, *J* = 5.4 Hz), 3.16 – 3.03 (m, 2H), 1.90 (p, 2H, *J* = 7.3 Hz), 1.38 – 1.22 (m, 8H), 0.99 (s, 3H), 0.91 (s, 3H), 0.90 – 0.85 (m, 3H).

**(*R*)-*N*-(2-(heptylsulfonamido)ethyl)-2,4-dihydroxy-3,3-dimethylbutanamide (79; CXP14.24-011)**

To a suspension of sodium heptane-1-sulfonate (100 mg, 0.494 mmol) in thionyl chloride (1 mL, 13.71 mmol) was added one drop of DMF. The mixture was heated to 75 °C and stirred for 16 h. Then, the mixture was allowed to cool to room temperature. The solids were removed by filtration and the filtrate was concentrated *in vacuo*. The residue was suspended in a mixture of MeCN (1 mL) and water (0.1 mL) and a solution of **50** (124 mg, 0.62 mmol) and DIPEA (0.24 mL, 1.38 mmol) in a mixture MeCN (1 mL) and water (0.1) mL was added dropwise. The formed suspension was stirred for 4 h. Then, the mixture was diluted with EtOAc (10 mL), sat. aq. NH_4_Cl (5 mL) and water (1 mL). The layers were mixed and separated and the aqueous phase was extracted with EtOAc (5 mL). The combined organic layers were washed with sat. aq. NaHCO_3_ (5 mL) and brine (5 mL), dried over Na_2_SO_4_ and concentrated *in vacuo*. The crude product was purified by flash column chromatography (EtOAc:MeOH = 99:1  95:5) affording **79** (84 mg, 48%) as a colourless oil. ^1^H NMR(CDCl_3_): 7.25 – 7.20 (m, 1H), 5.21 – 5.14 (m, 1H), 4.04 (s, 1H), 3.96 (br s, 1H), 3.57 – 3.47 (m, 3H), 3.47 – 3.37 (m, 1H), 3.33 – 3.25 (m, 2H), 3.14 (br s, 1H), 3.04 – 2.97 (m, 2H), 1.84 – 1.73 (m, 2H), 1.46 – 1.36 (m, 2H), 1.36 – 1.23 (m, 6H), 1.05 (s, 3H), 0.97 (s, 3H), 0.91 – 0.85 (m, 3H).

**CXP18.4-033** as described by Barnard *et al*. (20)
